# The Fine Tuning of Drp1-Dependent Mitochondrial Remodeling and Autophagy Controls Neuronal Differentiation

**DOI:** 10.3389/fncel.2019.00120

**Published:** 2019-04-04

**Authors:** Chiara Vantaggiato, Marianna Castelli, Matteo Giovarelli, Genny Orso, Maria Teresa Bassi, Emilio Clementi, Clara De Palma

**Affiliations:** ^1^Scientific Institute, IRCCS E. Medea, Laboratory of Molecular Biology, Lecco, Italy; ^2^Unit of Clinical Pharmacology, Department of Biomedical and Clinical Sciences “Luigi Sacco”, “Luigi Sacco” University Hospital, Università di Milano, Milan, Italy; ^3^Department of Pharmaceutical and Pharmacological Sciences, University of Padova, Milan, Italy; ^4^Unit of Clinical Pharmacology, “Luigi Sacco” University Hospital, ASST Fatebenefratelli Sacco, Milan, Italy

**Keywords:** Drp1, neuronal differentiation, mitochondrial remodeling, autophagy, mitochondrial fission

## Abstract

Mitochondria play a critical role in neuronal function and neurodegenerative disorders, including Alzheimer’s, Parkinson’s and Huntington diseases and amyotrophic lateral sclerosis, that show mitochondrial dysfunctions associated with excessive fission and increased levels of the fission protein dynamin-related protein 1 (Drp1). Our data demonstrate that Drp1 regulates the transcriptional program induced by retinoic acid (RA), leading to neuronal differentiation. When Drp1 was overexpressed, mitochondria underwent remodeling but failed to elongate and this enhanced autophagy and apoptosis. When Drp1 was blocked during differentiation by overexpressing the dominant negative form or was silenced, mitochondria maintained the same elongated shape, without remodeling and this increased cell death. The enhanced apoptosis, observed with both fragmented or elongated mitochondria, was associated with increased induction of unfolded protein response (UPR) and ER-associated degradation (ERAD) processes that finally affect neuronal differentiation. These findings suggest that physiological fission and mitochondrial remodeling, associated with early autophagy induction are essential for neuronal differentiation. We thus reveal the importance of mitochondrial changes to generate viable neurons and highlight that, rather than multiple parallel events, mitochondrial changes, autophagy and apoptosis proceed in a stepwise fashion during neuronal differentiation affecting the nuclear transcriptional program.

## Introduction

Mitochondrial dynamics and the balance between fusion and fission are key adaptative mechanisms to the metabolic needs of the cell (Gomes et al., 2011; Schrepfer and Scorrano, 2016). Recent evidence suggests that mitochondrial activity, shape and localization have an impact on nuclear programs and ultimately, cell fate. Therefore mitochondria are directly involved in differentiation and developmental processes (Kasahara and Scorrano, 2014). Mitochondria morphology, distribution and function are regulated by fusion and fission in response to the cellular environment and differentiation (Chang et al., 2006; Saxton and Hollenbeck, 2012). Three large GTPases are involved in mitochondrial fusion: Mitofusins 1 and 2 (Mfn1 and Mfn2), that mediate outer mitochondrial membrane fusion, and Optic atrophy 1 protein (Opa1), the mediator of inner-membrane fusion (Chan, 2012). Mitochondrial fission is coordinated by dynamin-related protein 1 (Drp1), mitochondrial adaptors, such as mitochondrial fission factor (Mff), the 49 and 51 kDa Mitochondrial Dynamics proteins (MiD49 and MiD51) and fission 1 (Fis1; Chan, 2012; Otera et al., 2013), and cytoplasmic elements. Among these, Drp1 is the major regulator of mitochondrial shape, distribution and maintenance (Reddy et al., 2011). Drp1 acts as a cytosolic receptor, linking fission and mitochondrial function to the cytoplasmic state of the cell. For instance changes in cytosolic Ca^2+^ levels activates the Ca^2+^-dependent phosphatase calcineurin enhancing Drp1 dephosphorylation on Ser637 and its translocation to mitochondria (Cereghetti et al., 2008). Conversely, cyclic AMP activates protein kinase A (PKA), resulting in inhibitory phosphorylation of Ser637 that blocks Drp1 translocation and promotes mitochondrial elongation (Cribbs and Strack, 2007).

Fission and fusion imbalance is a pathogenic mechanism common to many processes such as apoptosis, autophagy, aging and neurodegeneration and is correlated to myopathies, obesity, diabetes, cancer and neurodegenerative diseases (Wallace, 2005; Liesa et al., 2009; Itoh et al., 2013). The brain consumes about 20% of the body’s energy and considering its greatly metabolic activity, the delicate maintenance of mitochondrial function is essential for neurons (Chen and Chan, 2006; Kann and Kovács, 2007). As highly polarized cells, neurons require a suitable and appropriate distribution of mitochondria to provide energy fuel for neuronal activities, such as synaptic transmission, axonal and dendritic transport, and synaptic vesicle recycling (Li et al., 2004). This is allowed by mitochondrial fission and fusion that maintains cell bioenergetics and regulates mitochondria trafficking along the axons and at the synapses (Li et al., 2004, 2008; Chen and Chan, 2006). Mice lacking Drp1 die during embryogenesis and show neuronal development defects with depletion of mitochondria from neurites, reduced neurite outgrowth and impaired synapses formation (Ishihara et al., 2009; Wakabayashi et al., 2009; Kageyama et al., 2014). Drp1 and mitochondrial dynamics have also a role in the induction of pluripotent stem cells (iPSCs) from somatic cells (Xu et al., 2013; Wang et al., 2014; Prieto et al., 2016) and Drp1 depletion in mouse embryonic stem cells (ESCs) reduces the differentiation capacity to neurogenesis (Wang et al., 2014). Alterations in fission and fusion genes are associated with several neurodegenerative diseases: Mfn2 and Opa1 mutations are responsible for Charcot-Marie-Tooth (CMT) type 2A (Züchner et al., 2004) and dominant optic atrophy (DOA; Delettre et al., 2000) respectively, while Drp1 mutations have been associated with abnormal brain development (Waterham et al., 2007; Chang et al., 2010) and optic atrophy (Gerber et al., 2017).

Increased Drp1 expression and mitochondrial fragmentation are early and key events observed in a wide range of neurodegenerative disorders (Hu et al., 2017), including Alzheimer’s (Cho et al., 2009; Manczak et al., 2011; Manczak and Reddy, 2012), Huntington’s (Costa et al., 2010; Song et al., 2011; Shirendeb et al., 2012) and Parkinson’s diseases (Wang et al., 2011). Abnormal interactions between Drp1 and amyloid beta, phosphorylated Tau and Huntingtin proteins have also been reported (Wang et al., 2011; Manczak and Reddy, 2012). Increasing evidence indicates that mitochondrial dynamics also influence complex signaling pathways, affecting gene expression and cell differentiation (Kasahara and Scorrano, 2014), and that Drp1 and mitochondrial fission also plays a role in these processes. Indeed, migrating adult neural stem cells require appropriate Drp1 activity to maintain an efficient ATP synthesis and to differentiate properly (Kim et al., 2015). Moreover, NGF-induced neuronal differentiation is accompanied by higher Drp1 phosphorylation levels, early-upregulation of Opa1 and later induction of Mfn2, accounting for constant remodeling of mitochondria to suit morphological and functional changes of post-mitotic neurons (Martorana et al., 2018).

So far, the majority of the data available on Drp1 concerns the effects of the inhibition of mitochondrial fission by Drp1 silencing; considering the association between neurological disorders and high Drp1 levels we focused our study on the effects of increased levels of Drp1 on neuronal differentiation of P19 cells, a suitable model for the study of mammalian neuronal cell pathophysiology (Bain et al., 1994; Vantaggiato et al., 2009, 2011). In the presence of retinoic acid (RA) P19 cells form aggregates and differentiate into neurons and glia in a chronological order similar to that of cell differentiation in the brain. For this cell line an accurate protocol of neuronal differentiation was established (Bain et al., 1994) and we previously characterized morphological and biochemical markers specific for the different stages of the process (Vantaggiato et al., 2009, 2011). By genetically modulating Drp1, we found that the process of neuronal differentiation can be modified by changes in Drp1 levels and function, such that it is severely impaired by both Drp1 overexpression and depletion or inactivation. Increased fission or excessive fusion of mitochondria share similarities in the impairment of neuronal differentiation, inducing a transcriptional reprogramming that affects the response to RA with increased unfolded protein response (UPR) and ER-associated degradation (ERAD) pathways. This study also reveals that mitochondrial changes, autophagy and apoptosis proceed in a stepwise fashion and are permissive events for a correct neuronal differentiation.

## Materials and Methods

### Expression Constructs

pCMV6-MycDDK-Drp1wt and pCMV6-MycDDK-Drp1K38A vectors were purchased from OriGene Technologies, Inc., Rockville, MD, USA. The plasmids for RNA interference studies were purchased from SABiosciences Corporation (Frederick, MD, USA). A set of four different short-hairpin-RNAs (shRNAs) was tested (with neomycin selection or with the GFP reporter gene) and the most efficient one (shRNA1) was chosen. The shRNA specificity was determined by analysis of Drp1 expression levels in P19 cells transiently transfected with the shRNAs or with the scrambled control sequence by quantitative Real-Time PCR.

### Generation of Stable Clones

For neuronal differentiation experiments, P19 cells were stably transfected with pcDNA3.1/CTGFP vector alone, pCMV6-MycDDK-Drp1wt, pCMV6-MycDDK-Drp1K38A or Drp1shRNA plasmids, all with neomycin selection gene. Stable transfectants were obtained after selection in 500 μg/ml G418 (Invitrogen, Carlsbad, CA, USA, Thermo Fisher Scientific, Waltham, MA, USA) and Drp1 levels were analyzed by SDS-PAGE and Western Blot. The expression levels of endogenous Drp1 in the shRNA-transfected clones were determined by quantitative Real-Time PCR. For each vector, three different clones with comparable Drp1 levels were used independently in the experiments with similar results. Results shown for each vector are an average of the three clones, whereas limited to immunofluorescence images, we always reported representative pictures taken from the same clone.

### Cells Cultures and P19 Cells Neuronal Differentiation

The mouse embryo carcinoma P19 cells (Bain et al., 1994) were grown in Dulbecco’s Modified Eagle’s Medium (DMEM, Invitrogen, Carlsbad, CA, USA, Thermo Fisher Scientific, Waltham, MA, USA) supplemented with 10% Fetal bovine serum (FBS, Euroclone, Milano, Italy), 100 U/ml penicillin/streptomycin and 2 mM L-glutamine (Invitrogen, Carlsbad, CA, USA, Thermo Fisher Scientific, Waltham, MA, USA). P19 cells were differentiated into neurones and glial cells with 5 μM *all trans*-RA (Sigma-Aldrich, San Louis, MO, USA) for 4 days in bacteriological non-adhesive plates. Aggregates were then dissociated with trypsin, suspended in Neurobasal medium with N2 supplement (Invitrogen, Carlsbad, CA, USA, Thermo Fisher Scientific, Waltham, MA, USA) and 0.5 mM L-glutamine and plated onto 6-well plates coated with 100 μg/ml poly-L-lysine (Sigma-Aldrich, St. Louis, MO, USA), 1 × 10^6^ cells/well. For immunofluorescence, analysis cells were seeded on poly-L-lysine coated coverslip. Cells were cultured for a total of 14 days. Duplicate samples for each day were used for RNA or protein extraction.

### mRNA Levels Analysis

RNA was prepared using Trizol (Invitrogen, Carlsbad, CA, USA, Thermo Fisher Scientific, Waltham, MA, USA) and 1 μg/samples were reverse-transcribed into cDNA using the Superscript First Strand Synthesis System for RT-PCR kit (Invitrogen, Carlsbad, CA, USA, Thermo Fisher Scientific, Waltham, MA, USA) and random hexamers. The expression levels of Drp1, Mash1, Wnt1, Oct3/4, Sox2, Opa1, Mfn1, Mfn2, Fis1, MAP2, Tubulin beta III and Gap43 were analyzed by quantitative Real-Time PCR on an ABI PRISM^®^ 7900HT Fast Real-Time PCR Systems (Applied Biosystems, Foster City, CA, USA) using specific gene expression assays. TATA box binding protein (Tbp) was used for normalization. The expression levels of GP78, HRD1, GRP78, GRP94, CHOP and GADD34 were analyzed by quantitative Real Time PCR on the same instrument using specific primers. 36B4 was used for normalization. Primers used were: GRP78 F: 5′-TGTGGTACCCACCAAGAAGTC-3′ and R: 5′-TTCAGCTGTCACTCGGAGAAT-3′; GRP94 F: 5′-CTCAGAAGACGCAGAAGACTCA-3′ and R: 5′-AAAACTTCACATTCCCTCTCCA-3′; CHOP F: 5′-ATATCTCATCCCCAGGAAACG-3′ and R: 5′-TCTTCCTTGCTCTTCCTCCTC-3′; GADD34 F: 5′-GAGGGACGCCCACAACTTC-3′ and R: 5′-TTACCAGAGACAGGGGTAGGT-3′; GP78 F: 5′-AGCCTGTTCGTGTGGGTTC-3′ and R: 5′-AAATCTGTCTTTGCAGAGCTGAA-3′; HRD1F: 5′-CGTGTGGACTTTATGGAACGC-3′ and R: 5′-CGGGTCAGGATGCTGTGATAAG-3′; 36B4 F: 5′-AGATTCGGGATATGCTGTTGG 3′ and R: 5′-AAAGCCTGGAAGAAGGAGGTC-3′. Undifferentiated P19 cells stably transfected with the vector alone were used as endogenous control. Data were analyzed using the delta-delta-Ct method.

### Quantification of Mitochondrial DNA

Mitochondrial DNA (mtDNA) was quantified as described with slight modifications (Mouchiroud et al., 2013; De Palma et al., 2014). Total DNA was isolated from undifferentiated P19 cell clones using the QIAamp DNA mini kit (Sigma-Aldrich, St. Louis, MO, USA) according to the manufacturer’s instructions. The mtDNA content was measured by Real Time PCR by using specific primers for the not-polymorphic mitochondrial gene (NADH dehydrogenase 1, ND1). The single-copy nuclear gene RNAse P was used for normalization. Primers used were: mtND1 F: 5′-CCTATCACCCTTGCCATCAT-3′ and R: 5′-GAGGCTGTTGCTTGTGTGAC-3′; RNAse P F: 5′-GAAGGCTCTGCGCGGACTCG-3′ and R: 5′-CGAGAGACCGGAATGGGGCCT-3′. Undifferentiated P19 cells stably transfected with the vector alone were used as endogenous control. Data were analyzed using the delta-delta-Ct method.

### Mitochondria Isolation

For mitochondria isolation, undifferentiated and differentiated P19 cell clones were washed in PBS and suspended in ice-cold lysis buffer (0.3 M sucrose, 10 mM MES, 1 mM MgSO_4_, 1 mM KCl supplemented with proteases inhibitor cocktail from Sigma-Aldrich, St. Louis, MO, USA). Cells were sonicated and centrifuged at 1,500 rpm for 5 min at 4°C to pellet nuclei and cellular debris. Supernatants containing the mitochondrial fraction were collected and centrifuged at 10,000 rpm for 10 min at 4°C. Pellets were suspended in lysis buffer, assayed and subjected to SDS-PAGE and Western Blot. Voltage-dependent anion channels (VDAC) and GAPDH were used as mitochondrial and cytosolic markers, respectively.

### Immunoprecipitation

Cells were lysed in ice-cold lysis buffer (20 mM Tris-HCl pH 7.4, 150 mM NaCl, 0.1% Triton X-100, 10 mM MgCl_2_, 0.4 mM PMSF and protease inhibitor cocktail), sonicated and centrifuged at 13,000 rpm for 10 min at 4°C. Supernatants were assayed and an equal amount of total proteins for each cell line was immunoprecipitated with GTP-agarose beads (Sigma-Aldrich, St. Louis, MO, USA), that specifically binds GTP-bound proteins. Samples were rotated for 1 h at 4°C, immune-complexes were then washed three times with lysis buffer, eluted in 5× sample buffer plus 1 mM DTT at 95°C for 5 min and subjected to SDS-PAGE and Western Blot.

### SDS-PAGE and Western Blot

Cells were lysed in Tris-HCl 0.125 M pH 6.8 and 2.5% SDS, loaded on 10% or 12% polyacrylamide gel, blotted onto nitrocellulose membranes and probed with the indicated primary antibodies. Horseradish peroxidase-conjugated secondary antibodies were used and signals were detected using ECL (GE Healthcare, Chalfont St. Giles, UK).

### Antibodies

Antibodies (Abs) against MAP1LC3B, ERK1/2, phospho-ERK1/2, Akt, phospho-Akt, Smad1, phospho-Ser206-Smad1, GSK3β, phospho-Ser9-GSK3β, active caspase 7, active caspase 9, phospho-eIF2α and eukaryotic translation initiation factor 2 alpha (eIF2α) were purchased from Cell Signaling Technology, Inc. Danvers, MA, USA. Anti-Gap43 and β-actin Abs were purchased from Santa Cruz Biotechnology, Inc. Heidelberg, Germany. Anti-mitochondrial Cytochrome C Oxidase I (mtCO1), VDAC1, Tubulin β-III, MAP2 and Aconitase1 (ACO1) Abs were purchased from Abcam, Cambridge, UK. Anti-DDK Ab was purchased from OriGene Technologies. Anti-OPA1 and Drp1 Abs were purchased from Becton, Dickinson and Company, Franklin Lakes, NJ, USA. Anti-p62 and BNIP3 Abs were purchased from Sigma Aldrich.

### Confocal Immunofluorescence

Cells were fixed with 4% paraformaldehyde for 10 min and permeabilized with PBS containing 0.1% saponin and 1% bovine serum albumin for 30 min. Samples were then incubated for 2 h with primary Abs and revealed using the secondary Abs AlexaFluor-488, 546 and 647 (Invitrogen, Carlsbad, CA, USA, Thermo Fisher Scientific, Waltham, MA, USA). For the staining of autophagosomes, cells were transfected with the pCMVMAP1RFPLC3B vector (Addgene, Cambridge, MA, USA). For the staining of mitochondria, cells were transfected with the pDsRed2-Mito vector (Clontech Laboratories, Inc., CA, USA). Nuclei were counterstained with 4′,6′-diamidino-2-phenylindole (DAPI, Sigma-Aldrich, St. Louis, MO, USA). Images were acquired using a Leica TCS SP2 AOBS confocal laser scanning microscope with a 63× oil immersion lens at 1,024 × 1,024 pixels resolution, at the same laser attenuation.

### Mitochondria Morphometric Analysis

To quantify mitochondria morphology, an ImageJ macro was used (Dagda et al., 2009). The red channel of cells transfected with pDsRed2-Mito vector was extracted to grayscale, thresholded to optimally resolve individual mitochondria and converted to a binary image. The macro traces mitochondrial outlines using “analyze particles” and quantifies mitochondrial size, interconnectivity and elongation for each mitochondrion. The mitochondrial size was a measure of the area. The mean area/perimeter ratio was used as an index of mitochondrial interconnectivity, while inverse circularity was used as a measure of mitochondrial elongation. Interconnectivity describes the network of the mitochondria and higher scores for interconnectivity indicate that mitochondria have more physical connections, while lower scores indicate more fragmented mitochondria. Elongation describes the shape of mitochondria and a value of 1 would be considered a perfect circle, while a higher value represents elongated mitochondria. For the analysis of mitochondrial branching, the binary image was converted to a skeleton that represents the features in the original image, by using “skeletonize.” Finally, the length of each branch and the number of branches were determined by using the “analyze skeleton” plugin (Wiemerslage and Lee, 2016).

### Mitochondrial Membrane Potential Analysis

Mitochondria were labeled using tetramethylrhodamine methyl ester (TMRM; ThermoFisher Scientific, Waltham, MA, USA). Cells were seeded in 30 mm culture dish in complete medium and incubated with 100 nM TMRM and 1 μg/ml DAPI for 30 min at 37°C (Vega-Naredo et al., 2014). Images were acquired without replacing the medium, with a Spinning disk Nikon confocal microscope, with a 20× air lens and a 516 × 516 resolution, at the same laser attenuation. Fluorescence intensity was measured using the ImageJ programme as the average pixel intensity within a box of defined size drawn on the cell body. TMRM fluorescence was also determined by using a Fluoroskan (Ascent FL, ThermoFisher Scientific, Waltham, MA, USA). Cells were trypsinized, counted and incubated with 100 nM TMRM for 30 min at 37°C. Fluorescence was measured by using TRITC filter, accordingly to the manufacturer’s instruction. After background subtraction, the data were normalized on cell number.

### Mitochondria Respiratory Rate

Mitochondria respiratory rates were measured into the O2K oxygraph chambers (Oroboros Instruments, Innsbruck, Austria) at 37°C in the respiration medium MiR06 (0.5 mM EGTA, 3 mM MgCl_2_, 60 mM K-lactobionate, 20 mM taurine, 10 mM KH_2_PO_4_ 20 mM Hepes, 110 mM sucrose and 1 g/l bovine serum albumin fatty acid-free, 280 U/ml catalase (pH 7.1)).

Drp1 clones were grown in standard conditions and 1 × 10^6^ viable cells were transferred into oxygraph chambers. Oxygen consumption reached a steady state level indicating Basal respiration. The addition of oligomycin (0.5 μM) resulted in Leak respiration. Subsequently, the mitochondrial proton gradient was lost by stepwise titration (0.5 μM each step) of the uncoupler FCCP (Carbonyl cyanide-*4*-(trifluoromethoxy) phenylhydrazone) until the maximum respiration was achieved. The addition of 0.5 μM rotenone and 2.5 μM antimycin A (AA) blocked mitochondrial respiration, showing residual oxygen consumption. The initial addition of pyruvate (10 mM) and malate (2 mM) was performed to test the integrity of the plasma membrane. Oxygen fluxes were corrected by subtracting residual oxygen consumption from each steady state.

### Cell Death Analysis

Control, Drp1wt, Drp1K38A and Drp1shRNA P19 stable clones were induced to differentiate with RA and cell death was analyzed 24 h later using propidium iodide (PI) and DAPI (Hamada-Kanazawa et al., 2004). Cell aggregates were incubated with 3 μM PI (Sigma-Aldrich, St. Louis, MO, USA) and 1 μg/ml DAPI for 15 min at room temperature to stain respectively nuclei of dead and living cells and photographed using a Leica DMIRE2 microscope at 20× magnification and 1,024 × 1,024 pixels resolution. Apoptosis on d1 of neuronal differentiation was quantified by using a DeadEnd Fluorometric TUNEL system kit (Promega, Madison, WI, USA), accordingly with manufacturer’s instructions. Briefly, on d1 aggregates were dissociated, counted, fixed in 1% paraformaldehyde for 20 min on ice and permeabilized with PBS containing 0.2% TRITON-X100 for 10 min. Cells were then incubated with the Terminal Deoxynucleotidyl Transferase enzyme at 37°C. Nuclei were counterstained with 1 μg/ml DAPI. TUNEL fluorescence was quantified by using a Fluoroskan with FITC filter. After background subtraction, the data were normalized on cell number.

Apoptosis was detected in the same cells treated with RA after aggregates dissociation on day 5 and 6 of neuronal differentiation using a Caspase 3 Colorimetric Activity Assay kit (Merck Millipore, Burlington, MA, USA), according to the manufacturer’s instructions.

### Statistical Analysis

**Figure 1:**
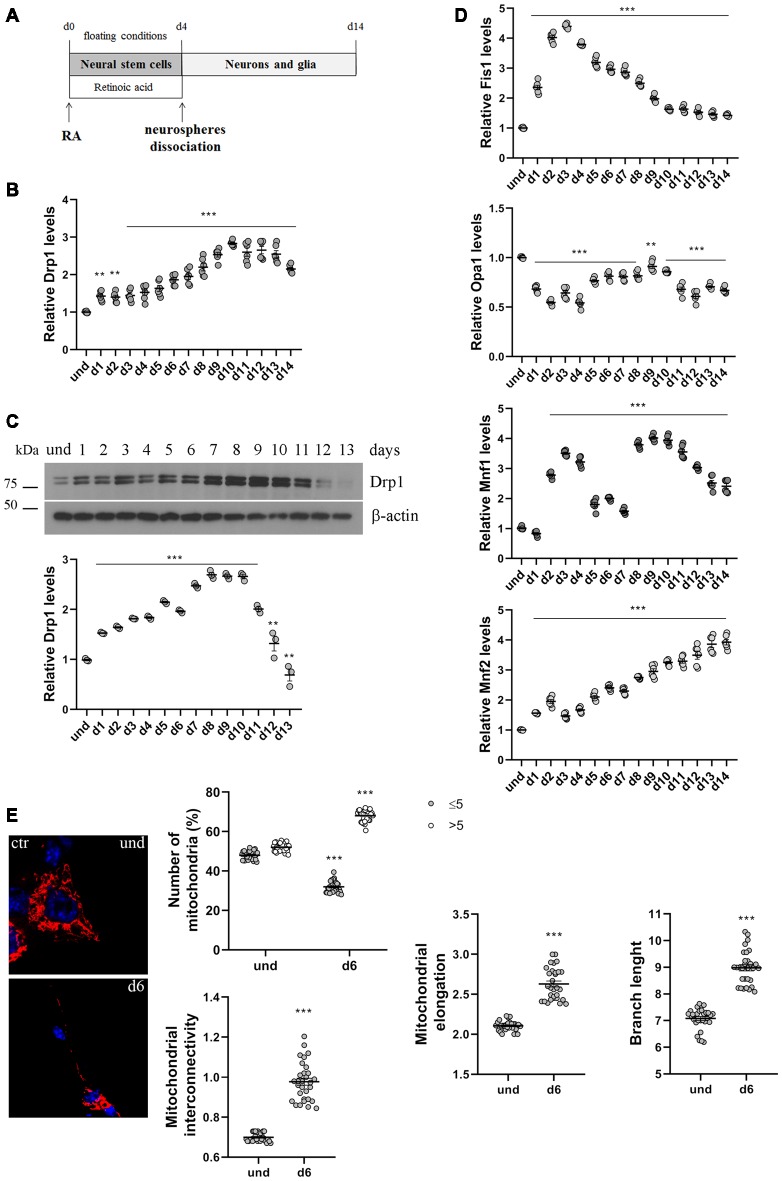
P19 cells neuronal differentiation. **(A)** Schematic representation of P19 cells neuronal differentiation. Cells were incubated with retinoic acid (RA) for 4 days in floating conditions to induce the formation of neurospheres and the differentiation in neural stem cells. On d4 neurospheres were dissociated and plated in adherent conditions to differentiate in neurons and glia. **(B)** Analysis of Drp1 expression levels during neuronal differentiation. P19 cells were induced to differentiate with RA and RNA was extracted every day from d1 to d14 and used to analyze Drp1 expression levels by Real-Time PCR. Results are expressed as fold increase of undifferentiated control cells, used as endogenous control, as individual data plus the mean ± standard error of the mean (SEM) (one-way ANOVA followed by Dunnett’s multiple comparison test, *n* = 6). **(C)** Analysis of Drp1 protein levels during neuronal differentiation. P19 cells were induced to differentiate with RA and total extracts were prepared every day, run on a 10% SDS-polyacrylamide gel and probed with anti Drp1 and actin Abs. Drp1 levels were quantified, normalized on actin levels and expressed as fold increase of undifferentiated cells. The graph shows individual data plus the mean ± SEM (one-way ANOVA followed by Dunnett’s multiple comparison test, *n* = 6). Uncropped gels are inSupplementary Figure S1. **(D)** Analysis of fission and fusion genes expression levels during neuronal differentiation. RNA extracted every day of neuronal differentiation was used to analyze Opa1, Mfn1, Mfn2 and Fis1 expression levels by Real-Time PCR. Results are expressed as fold increase of undifferentiated control cells, used as endogenous control, as individual data plus the mean ± SEM (one-way ANOVA followed by Dunnett’s multiple comparison test, *n* = 6). **(E)** Mitochondrial morphology in undifferentiated and differentiated P19 cells. Undifferentiated cells and neurons on d5 were transfected with the pDsRed2-Mito vector for the staining of mitochondria and fixed after 24 h. Nuclei were stained with DAPI. Image was acquired by confocal microscopy and morphometric analysis was performed with ImageJ. Red channels were converted into a black binary image and skeletonized (binary and skeleton images are in Supplementary Figure S2). Mitochondrial interconnectivity, elongation and branch length are showed in the graphs as individual data plus the mean ± SEM (unpaired *t*-test). Mitochondrial interconnectivity is a measure of physical connections and low scores indicate fragmented mitochondria; mitochondrial elongation describes the shape of mitochondria and a value of 1 would be considered a perfect circle, while higher value represents elongated mitochondria. The number of fragmented or elongated mitochondria, with an area lower or higher than 5 μm^2^ respectively, was also determined and reported in the graph as individual data plus the mean ± SEM (two-way ANOVA followed by Sidak’s multiple comparison test). Data were obtained from three independent experiments for a total of at least 30 cells for each sample. * vs und (***p < 0.001; **p < 0.01).

One-way or two way ANOVA followed by Sidak’s, Dunnett’s or Tukey’s multiple comparison test were performed using GraphPad Prism version 8.0.1 for Windows, GraphPad Software, San Diego, California USA^1^. Student’s *t*-test for unpaired variables (two-tailed) was used for mitochondrial elongation, interconnectivity and branch length control data analysis of Figure 1E. Results are reported as individual data plus the mean and standard error of the mean (SEM). *p* values of less than 0.05 were considered significant. Double or triple symbols refer to statistical probabilities (*p* < 0.01 and <0.001, respectively), measured in the various experimental conditions as detailed in the legend of the figures.

## Results

### Drp1 and Mitochondrial Remodeling Are Involved in Neuronal Differentiation

We first analyzed changes in Drp1 levels and mitochondrial morphology during neuronal differentiation. We incubated P19 cells with RA for 4 days in floating conditions to induce the formation of neurospheres and neural stem cells that differentiate into neurons after dissociation and plating in adherent conditions on d4 (Figure 1A). We found that Drp1 expression levels gradually increased in neural stem cells during RA treatment to rich 2.5–3-fold increase in differentiated neurons (d9-d10; Figures 1B,C and Supplementary Figure S1), suggesting that the regulation of Drp1 levels could be a key event during neuronal differentiation. Moreover, we found that P19 cells neuronal differentiation is characterized by changes in the expression levels of other fission and fusion genes (Figure 1D). Indeed, the expression of the fission gene Fis1 increased in neural stem cells with a peak between d2 and d4 to decrease thereafter. Opa1 decreased after RA addition and remained low or similar to basal for the whole differentiation process. Mfn2 levels increased during neuronal differentiation quadrupling its expression in the later stages (Figure 1D). Finally, Mfn1 had a bimodal expression pattern with a peak in the early phase of differentiation around d3 and later on d9.

To assess mitochondrial morphology, cells were transfected with pDsRed2-Mito vector and mitochondrial size, interconnectivity, elongation and branch length were quantified (Figure 1E and Supplementary Figure S2). We found that differentiated neurons on d6 presented filamentous mitochondria with increased interconnectivity, elongation and branch length compared with undifferentiated cells. Moreover, while undifferentiated cells presented a mixed population of fragmented and elongated mitochondria, differentiated neurons showed a decrease in the percentage of fragmented mitochondria and an increase in the elongated one (Figure 1E), confirming changes in mitochondrial morphology observed between undifferentiated P19 cells and neural stem cells on d4 (Vega-Naredo et al., 2014).

### Different Expression of Drp1 Impacts on Mitochondrial Morphology and Functionality

**Figure 2:**
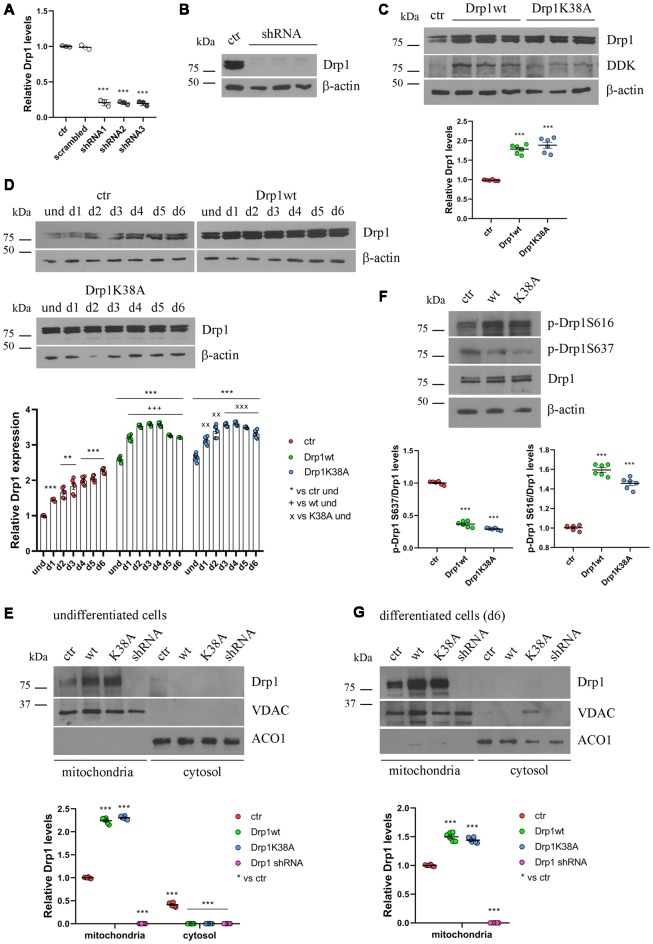
Drp1 levels and localization in Drp1-modified clones. **(A)** Drp1 expression levels in Drp1 shRNA stable clones. RNA was extracted from P19 clones stably transfected with Drp1 shRNA. Drp1 expression levels were analyzed by Real Time PCR and compared with Drp1 levels in P19 transfected with the scrambled sequence. P19 cells stably transfected with the vector alone were used as endogenous control (ctr, set at 1). The graph shows the residual Drp1 expression in the three Drp1 shRNA clones used in the experiments. Results are expressed as individual data plus the mean ± SEM (one-way ANOVA followed by Dunnett’s multiple comparison test, *n* = 3). * vs ctr (*** *p* < 0.001). **(B)** Drp1 protein levels in Drp1 shRNA stable clones. Total extracts prepared from the three Drp1 shRNA clones selected for the experiments were run on a 10% gel and probed with anti Drp1. Uncropped gels are in Supplementary Figure S3. **(C)** Drp1 protein levels in Drp1wt and Drp1K38A overexpressing clones. Total extracts prepared from the Drp1wt and Drp1K38A stable clones selected for the experiments, were run on 10% gel and probed with anti Drp1, DDK and actin Abs. Drp1 levels were quantified, normalized on actin levels and expressed as fold increase of control. The graph shows individual data plus the mean ± SEM (one-way ANOVA followed by Dunnett’s multiple comparison test, *n* = 6). * vs ctr (***p < 0.001). Uncropped gels are in Supplementary Figure S3. **(D)** Drp1 expression levels increase during Drp1wt and Drp1K38A stable clones neuronal differentiation. Total extracts from undifferentiated Drp1wt, Drp1K38A and control clones and from d1 to d6 of neuronal differentiation, were run on a 10% SDS-polyacrylamide gel and probed with anti Drp1 and actin Abs. Drp1 levels were quantified, normalized on actin levels and expressed as fold increase of undifferentiated control levels. The graph shows individual data plus the mean ± SEM (two-way ANOVA followed by Tukey’s multiple comparison test, *n* = 6). * vs ctr und (***p < 0.001, **p < 0.01); + vs Drp1wt und (^+++^*p* < 0.001); × vs Drp1K38A und (^xxx^*p* < 0.001, ^xx^*p* < 0.01). Uncropped gels are in Supplementary Figure S3. **(E)** Localization of Drp1 on mitochondria in undifferentiated Drp1-modified clones. Mitochondrial and cytosolic extracts were prepared from undifferentiated Drp1wt, Drp1K38A, Drp1 shRNA and control clones, run on 10% SDS-polyacrylamide gels and probed with anti Drp1, VDAC and ACO1 Abs. VDAC and ACO1 were used as mitochondrial and cytosolic marker respectively. Drp1 levels were quantified, normalized on VDAC levels and expressed as fold increase of control. The graph shows individual data plus the mean ± SEM (two-way ANOVA followed by Tukey’s multiple comparison test, *n* = 6). * vs ctr (***p < 0.001). Uncropped gels are in Supplementary Figure S5. **(F)** Drp1 phosphorylation levels in undifferentiated Drp1wt and Drp1K38A clones. Total extracts from Drp1wt, Drp1K38A and control clones were run on 10% SDS-polyacrylamide gels and probed with anti Drp1, phospho-Drp1 Ser616, phospho-Drp1 Ser637 and actin Abs. The phosphorylation level of Ser616 and Ser637 were quantified, normalized on total Drp1 levels and expressed as fold increase of control. The graph shows individual data plus the mean ± SEM (one-way ANOVA followed by Dunnett’s multiple comparison test, *n* = 6). * vs ctr (***p < 0.001). Uncropped gels are in Supplementary Figure S3. **(G)** Localization of Drp1 on mitochondria in Drp1-modified clones-derived neurons. Mitochondrial and cytosolic extracts were prepared from Drp1wt, Drp1K38A, Drp1 shRNA and control clones on d6 of neuronal differentiation and processed as in **(E)**. Drp1 levels were normalized on VDAC levels and expressed as fold increase of control. The graph shows individual data plus the mean ± SEM (one-way ANOVA followed by Dunnett’s multiple comparison test, *n* = 6). * vs ctr (***p < 0.001). Uncropped gels are in Supplementary Figure S5.

We then studied how altered Drp1 levels and increased fragmentation could affect neuronal differentiation. To this purpose, we stably transfected P19 cells with wild-type Drp1 (Drp1wt), the dominant negative mutated form (Drp1K38A), a specific shRNA to silence the gene (Drp1 shRNA) or an empty vector as control. Three clones for each vector with comparable Drp1 expression levels were selected for the experiments. The selected Drp1-silenced clones showed a reduction of 80% in Drp1 expression levels (Figures 2A,B), while Drp1 was two-fold increase in Drp1wt and Drp1K38A selected clones (Figure 2C and Supplementary Figure S3).

Neuronal differentiation in control cells was accompanied by a progressive increase in Drp1 levels (Figure 1B); surprisingly this also occurred in Drp1wt and Drp1K38A clones (Figure 2D). By contrast, changes in Drp1 levels were not associated with changes in the expression of the other fission and fusion genes, Fis1, Mnf1, Mnf2 and Opa1, in both undifferentiated and differentiated cells (Supplementary Figures S4A,B), nor in Opa1 activity (Supplementary Figures S4C,D), excluding any compensatory activity.

Next, we analyzed the effect of Drp1wt, Drp1K38A overexpression and gene silencing on mitochondrial morphology and functionality during neuronal differentiation. Drp1 mostly localizes into the cytoplasm and is recruited to mitochondria membrane to induce fission (Ingerman et al., 2005). We quantified the amount of Drp1 in the mitochondrial fraction in undifferentiated cells and we found that while in control cells 60% of Drp1 was localized in the mitochondria and the remaining 40% was in the cytosol, in Drp1wt and Drp1K38A overexpressing clones Drp1 showed almost a complete translocation on mitochondria (Figure 2E and Supplementary Figure S5). This mitochondrial recruitment correlated with an increase in Ser616 and a decrease in Ser637 phosphorylation levels in both clones compared with control (Figure 2F), a condition that induces Drp1 translocation (Otera et al., 2013). Moreover, we found that neuronal differentiation affected Drp1 translocation on mitochondria *per se*. Indeed, on d6 of neuronal differentiation control cells displayed a complete localization of Drp1 on mitochondria (Figure 2G and Supplementary Figure S5), while no differences were observed in Drp1-modified clones, showing a complete Drp1 translocation in both undifferentiated and differentiated conditions (Figures 2E,G).

**Figure 3:**
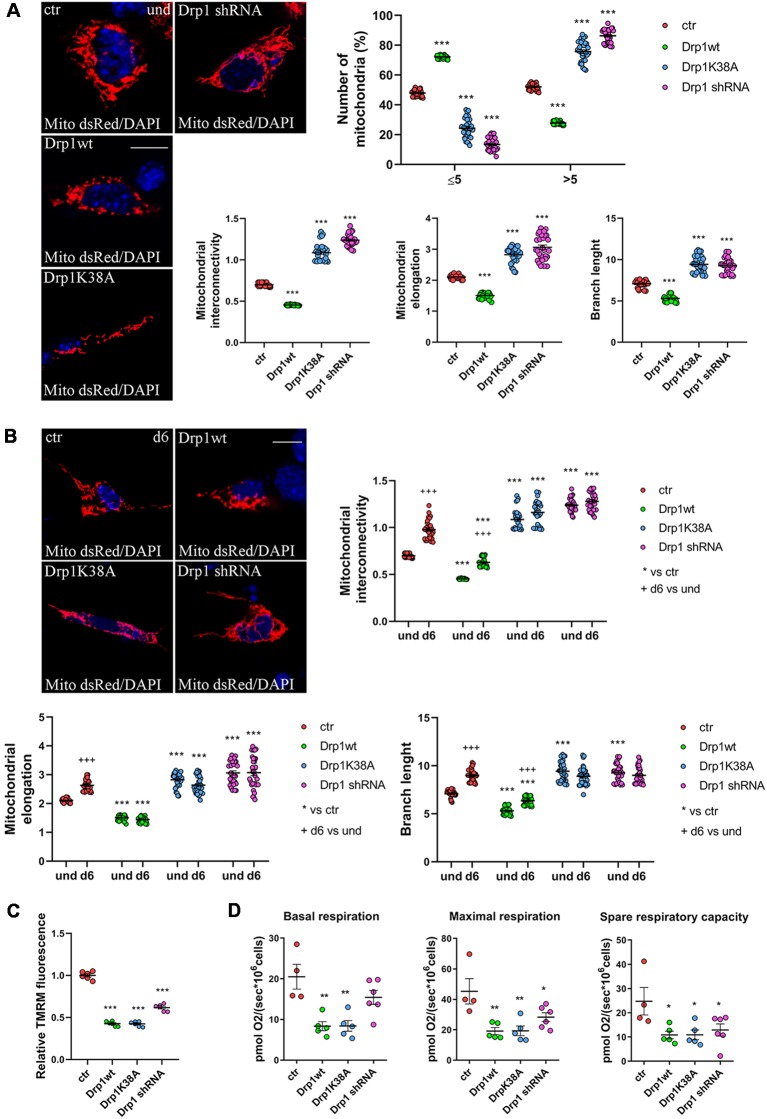
Mitochondrial morphology. **(A)** Mitochondrial morphology in undifferentiated Drp1-modified clones. Drp1wt, Drp1K38A, Drp1 shRNA and control cells were transfected with the pDsRed2-Mito vector for the staining of mitochondria and fixed after 24 h. Nuclei were stained with DAPI. Images were acquired by confocal microscopy and morphometric analysis was performed with ImageJ. Red channels were converted into a black binary image and skeletonized (binary and skeleton images are in Supplementary Figure S6A). Mitochondrial interconnectivity, elongation and branch length are showed in the graphs as individual data plus the mean ± SEM (one-way ANOVA followed by Dunnett’s multiple comparison test). The number of fragmented or elongated mitochondria, with an area lower or higher than 5 μm^2^ respectively, was also determined and reported in the graph. Results are expressed as individual data plus the mean ± SEM (two-way ANOVA followed by Sidak’s multiple comparison test). Data were obtained from three independent experiments for a total of at least 30 cells for each sample. Scale bar: 10 μm. * vs ctr (*** *p* < 0.001). **(B)** Mitochondrial morphology in differentiated neurons (d6). Drp1wt, Drp1K38A, Drp1 shRNA and control clones were induced to differentiate with RA, on d5 neurons were transfected with the pDsRed2-Mito vector and processed as described in **(A)**. Binary and skeleton images are in Supplementary Figure S6B. Mitochondrial interconnectivity, elongation and branch length are shown in the graphs. Results are expressed as individual data plus the mean ± SEM (two-way ANOVA followed by Sidak’s multiple comparison test). * vs ctr (***p < 0.001); ^+^d6 vs und (^+++^*p* < 0.001). Data were obtained from three independent experiments for a total of at least 30 cells for each sample. Scale bar: 10 μm. **(C)** Mitochondrial membrane potential. Undifferentiated cells were trypsinized, counted and incubated with 100 nM TMRM for 30 min at 37°C. Fluorescence was measured with a Fluoroskan by using a TRITC filter. After background subtraction, the data were normalized on cell number and reported as fold increase of control. Results are expressed as individual data plus the mean ± SEM (one-way ANOVA followed by Tukey’s multiple comparison test, *n* = 6). * vs ctr (***p < 0.001). **(D)** Oxygen consumption measurement on intact undifferentiated clones. Basal and maximal respiratory capacity are reported while spare respiratory capacity was obtained by subtracting basal respiration from maximal respiration rates. Results show individual data plus the mean ± SEM (one-way ANOVA followed by Dunnett’s multiple comparison test, *n* = 4–6). * vs ctr (**p < 0.01, *p < 0.05).

We next assessed mitochondrial morphology by staining mitochondria with pDsRed2-Mito vector and quantifying mitochondrial size, interconnectivity, elongation, branch length and the number of fragmented and elongated mitochondria (Figure 3A and Supplementary Figure S6A). In undifferentiated conditions, Drp1wt overexpressing clones displayed mitochondrial fragmentation with a higher percentage of small round mitochondria (70%) compared with control (40%) and decreased elongation, interconnectivity and branch length (Figure 3A), in agreement with the increased translocation of Drp1 on mitochondria. Conversely, Drp1-silenced and Drp1K38A-expressing clones presented filamentous mitochondria, characterized by increased mitochondrial elongation, interconnectivity and branch length compared with control (Figure 3A). When we analyzed mitochondrial remodeling during neuronal differentiation we found that mitochondria in differentiated Drp1wt overexpressing clones (d6) presented unchanged elongation but increased interconnectivity and branch length, compared to undifferentiated conditions (d6 vs. und), however, these parameters were still significantly reduced compared to control (Figure 3B and Supplementary Figure S6B). This indicates that neuronal differentiation activates mitochondrial remodeling in Drp1wt clones, but fails to induce the correct and required elongation. Surprisingly, in Drp1K38A and Drp1-silenced clones mitochondria maintained the same shape in differentiated and undifferentiated conditions, without remodeling (Figure 3B, d6 vs. und).

Finally, we investigated mitochondria functionality with TMRM, a red fluorescent dye that is sequestered by active mitochondria, to assess mitochondrial membrane potential. All clones were incubated with TMRM and fluorescence intensity was captured in live cells by confocal microscopy (Supplementary Figure S7) and quantified by Fluoroskan (Figure 3C). Of note, all Drp1-modified clones presented a decrease in TMRM fluorescence compared with control, indicating low mitochondria membrane potential and likely poor functionality, regardless of morphology. To further assess whether the mitochondrial metabolism was impaired we measured oxygen consumption in undifferentiated clones (Figure 3D). In agreement with TMRM analysis, basal mitochondrial respiration was impaired in Drp1wt and Drp1K38A clones compared to control and all Drp1-modified clones were not able to sustain the increased workload driven by the addition of the protonophore FCCP. Consistently, spare respiratory capacity calculated by subtracting basal respiration from FCCP-induced respiration was reduced in all clones, indicating that the alterations of Drp1 levels/activity affect the capability of the respiratory chain to match an energetic request, as well as the maximal respiration achievable by the cells. These data confirm a mitochondrial bioenergetic defect regardless of mitochondrial morphology.

### Different Expression of Drp1 Impacts on Mitophagy

**Figure 4:**
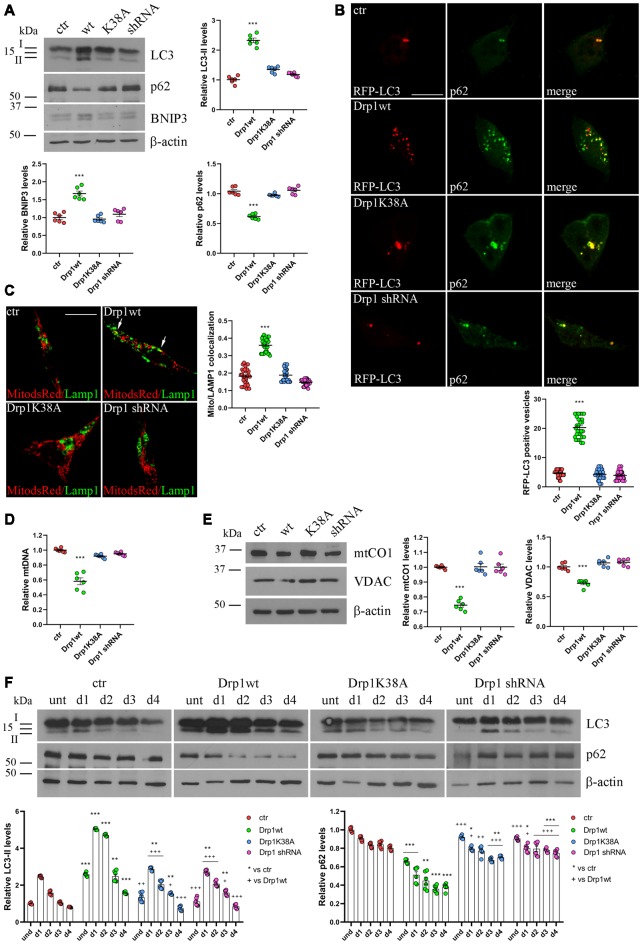
Mitochondrial biogenesis and autophagy in Drp1-modified clones. **(A)** Autophagy is altered in clones overexpressing Drp1wt. Total extracts from Drp1wt, Drp1K38A, Drp1 shRNA and control cells were run onto 12% or 10% SDS-polyacrylamide gels and probed with anti MAP1LC3B, p62, BNIP3 and actin Abs. The two isoforms LC3-I and LC3-II are indicated. LC3-II, p62 and BNIP3 levels were quantified, normalized on actin levels and expressed as fold increase of control. The graphs show individual data plus the mean ± SEM (one-way ANOVA followed by Dunnett’s multiple comparison test, *n* = 6). Uncropped gels are in Supplementary Figure S8. **(B)** Drp1wt, Drp1K38A, Drp1 shRNA and control cells were transfected with MAP1LC3B-RFP for the staining of autophagosomes (red), fixed 24 h later and immunostained with anti p62 (green) Ab. Yellow in the merge images indicates colocalization of p62 and RFP-LC3. Total RFP-LC3 positive vesicles number is shown in the graph as individual data plus the mean ± SEM (one-way ANOVA followed by Dunnett’s multiple comparison test). Data were obtained from three independent experiments for a total of at least 30 cells for each sample. Scale bar: 10 μm. **(C)** Mitophagy is altered in clones overexpressing Drp1wt. Drp1wt, Drp1K38A, Drp1 shRNA and control clones were transfected with pDsRed2-Mito vector for the staining of mitochondria (red), fixed 24 h later and immunostained with anti Lamp1 (green). Yellow indicates co-localization. Pearson’s correlation coefficients for Mito dsRed and Lamp1 colocalization were determined in at least 30 cells/staining and reported in the graph as individual data plus the mean ± SEM (one-way ANOVA followed by Dunnett’s multiple comparison test). Scale bar: 10 μm. **(D)** Drp1wt overexpressing clones show reduced mitochondrial DNA (mtDNA). DNA was extracted from undifferentiated clones and used to quantify mtDNA by Real-Time PCR by using specific primers for NADH dehydrogenase 1 (ND1). RNAse P was used for normalization. Results are expressed as fold increase of control. Graph shows individual data plus the mean ± SEM (one-way ANOVA followed by Dunnett’s multiple comparison test, *n* = 6). **(E)** Drp1wt overexpressing clones show reduced mitochondrial content. Total extracts were prepared from undifferentiated clones, run on 10% SDS-polyacrylamide gels and probed with anti mtCO1 and VDAC Abs. mtCO1 and VDAC levels were quantified and normalized on actin levels. Results are expressed as fold increase of control individual data plus the mean ± SEM (one-way ANOVA followed by Dunnett’s multiple comparison test, *n* = 6). Uncropped gels are inSupplementary Figure S8. **(F)** Autophagy levels in neural stem cells aggregates. Drp1wt, Drp1K38A, Drp1 shRNA and control clones were induced to differentiate with RA and total protein extracts were prepared each day from d1 to d4, run onto 15% or 10% SDS-polyacrylamide gels and probed with anti MAP1LC3B, p62 and actin Abs. LC3-II and p62 levels were quantified, normalized on actin levels and expressed as fold increase of control. The graphs show individual data plus the mean ± SEM (two-way ANOVA followed by Tukey’s multiple comparison test, *n* = 6). Lines indicate samples to whom symbols are referred. Uncropped gels are in Supplementary Figure S8. * vs ctr (***p < 0.001; **p < 0.01; *p < 0.05); ^+^vs Drp1wt (^+++^*p* < 0.001; ^++^*p* < 0.01; ^+^*p* < 0.05).

Dysfunctional mitochondria are removed by mitophagy, allowing their selective degradation. To monitor the activation of mitophagy in our clones we evaluated the autophagosomal marker LC3 and SQSTM1/p62, a protein targeting poly-ubiquitinated proteins to autophagosomes for degradation. During autophagosome formation the cytosolic LC3-I isoform is converted into LC3-II and is incorporated in the autophagosome membrane, thus LC3-II amount correlates with the number of autophagosomes (Kabeya et al., 2000). In undifferentiated Drp1-silenced and Drp1K38A clones, LC3-II and p62 levels were similar to control, while Drp1wt overexpressing clones showed increased LC3-II and reduced p62 levels, indicating increased autophagy (Figure 4A and Supplementary Figure S8). Consistently, the number of RFP-LC3 positive autophagosomes was enhanced in Drp1wt overexpressing clones compared with control (Figure 4B).

Moreover, BNIP3, a potent inducer of mitophagy (Lee et al., 2011), raised in Drp1wt overexpressing clones (Figure 4A) and the sequestration of mitochondria into lysosomes, measured by colocalization between the mitochondrial marker Mito-dsRed and the lysosomal marker Lamp1 (Figure 4C), was evident in these clones, clearly highlighting mitophagy activation. No differences were observed between control and Drp1 silenced clones, confirming previous data (Kageyama et al., 2014). In agreement with these findings, Drp1wt overexpressing clones showed a reduction in mtDNA content compared with control, Drp1-silenced and Drp1K38A clones (Figure 4D). Accordingly, the levels of two mitochondrial proteins, mtCO1 and VDAC, were lower in undifferentiated Drp1wt overexpressing cells, confirming the reduction of mitochondrial mass only in this clone (Figure 4E).

We then analyzed autophagy during neuronal differentiation. Autophagy plays a role in the first days of neuronal differentiation removing old material and providing recycled constituents for building up new structures (Guan et al., 2013). As expected, autophagy in control cells enhanced at d1 during neurogenesis and decreased during the time (Figure 4F). The pattern of autophagy induction was similar in all clones, but with the highest levels in Drp1wt overexpressing clones (Figure 4F), confirming that high Drp1 levels impact on both basal and stimulated autophagy, enhancing the process.

### Physiological Levels of Drp1 Are Required for the Expression of Neurogenic Transcription Factors and the Downregulation of Pluripotency Genes, Preventing Apoptosis

**Figure 5:**
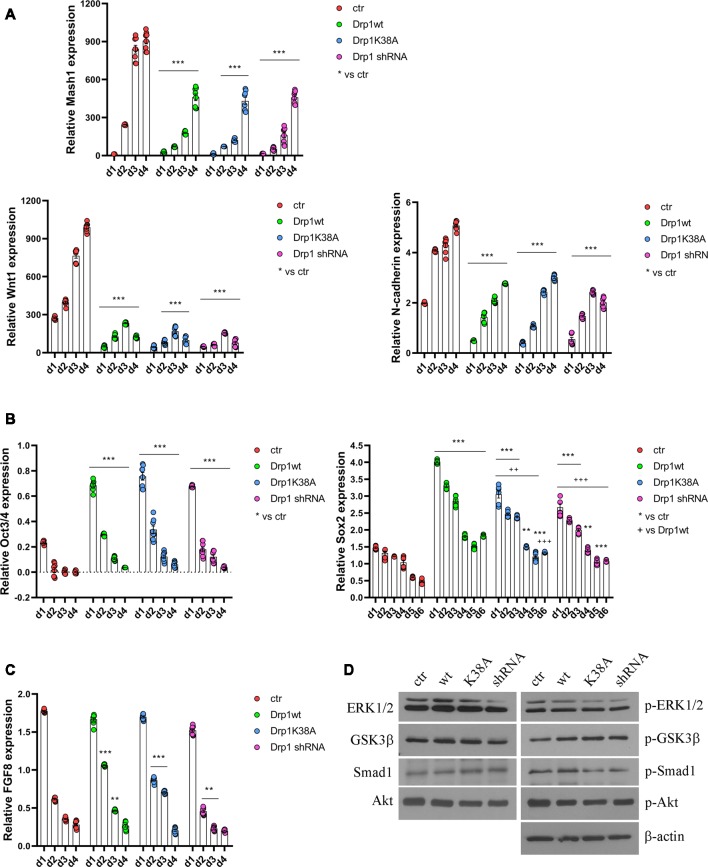
Alterations in Drp1 levels and function affect neuronal differentiation induction. Drp1wt, Drp1K38A, Drp1 shRNA and control P19 stable clones were induced to differentiate with RA. RNA was extracted from d1 to d6 and used to analyze Mash1, Wnt1 and N-cadherin (d1–d4; **A**), Oct3/4 (d1–d4) and Sox2 (d1–d6) **(B)**, and FGF8 (d1–d4) expression levels **(C)** by Real-Time PCR. Undifferentiated P19 cells stably transfected with the vector alone were used as an endogenous control (set at 1). Results are expressed as fold increase of endogenous control and reported as individual data plus the mean ± SEM (two-way ANOVA followed by Tukey’s multiple comparison test, *n* = 8). * vs ctr (***p < 0.001, **p < 0.01); ^+^ vs Drp1wt (^+++^*p* < 0.001, ^++^*p* < 0.01). Lines indicate samples to whom asterisks are referred. **(D)** Total extracts were prepared from Drp1wt, Drp1K38A, Drp1 shRNA and control clones 24 h after RA addiction, run on a 10% SDS-PAGE gel and probed with anti phospho-Akt (p-Akt), Akt, phospho-Ser9-GSK3β (p-GSK3β), GSK3β, phospho-ERK1/2 (p-ERK), ERK1/2, phospho-Ser206-Smad1 (p-Smad1) and Smad1 Abs. Uncropped gels are in Supplementary Figure S10. Quantification is reported in Supplementary Figure S11.

All clones were induced to differentiate with RA and formed neurospheres similar in number and size, thus on d4 they were dissociated, plated and cultured for several days. We started our analysis from the first phases of neuronal differentiation corresponding to the expression of the RA-induced neurogenic transcription factors (Bain et al., 1994; Vantaggiato et al., 2011). We found that, while in control cells RA treatment induced the expression of Mash1, Wnt1 and N-cadherin (Figure 5A), their levels were significantly reduced in all Drp1-modified clones, indicating that any manipulation of Drp1 affected RA-induced transcription. Neuronal differentiation is also defined by the loosing of pluripotency through the downregulation of Oct3/4 and Sox2 genes (Li et al., 2013). While in control cells Oct3/4 expression levels were dramatically reduced by RA treatment starting from d1 to disappear on d2, accordingly with previous data (Bain et al., 1994; Yamada et al., 2013), in all Drp1-modified clones Oct3/4 levels were higher than control on d1 and residual levels were still present on d4 (Figure 5B). Sox2 is a neural stem cells marker and maintains neuronal progenitor identity (Graham et al., 2003), therefore its expression levels slightly increase on d1 of neuronal differentiation and slowly decrease during the next 4 days to reach the lowest levels in differentiated neurones (d5–d7; Yamada et al., 2013), as we observed in control cells (Figure 5B). By contrast, all Drp1-modified clones presented increased Sox2 levels on d1 of neuronal differentiation, that remained higher also on d5 and d6 (Figure 5B). On the contrary, no differences in Oct3/4 and Sox2 expression were observed among undifferentiated Drp1-modified and control clones (Supplementary Figure S9), indicating that Drp1 is not necessary for the maintenance of pluripotency in undifferentiated cells, but it is required for pluripotency genes downregulation during neuronal differentiation.

In parallel to its role in neuronal differentiation induction, RA, together with BMP4, is involved also in apoptosis, through the induction of caspase 9 and caspase 3 (Miho et al., 1999). The fate between neuronal differentiation and cell death depends on a fine regulation of the pro-apoptotic RA/BMP4 signaling and the pro-survival FGF8 pathway (Massagué, 1998; Vantaggiato et al., 2011). We analyzed FGF8 levels and its activated pathways, i.e., PI3K/Akt and ERK1/2 MAPK (Wang et al., 2006; Vantaggiato et al., 2011), during neuronal differentiation and we found that FGF8 expression levels (Figure 5C) as well as Akt, ERK1/2 and GSK3β-Ser9 phosphorylation levels had the same trend in all clones compared to control (Figure 5D, Supplementary Figures S10, S11). Also, the ERK1/2-dependent inhibitory phosphorylation on Ser206 of Smad1, a BMP4-activated protein that induces the expression of BMP target genes (Pera et al., 2003), was similar in Drp1-modified and control clones, indicating a correct inhibition of the BMP4 pathway.

**Figure 6:**
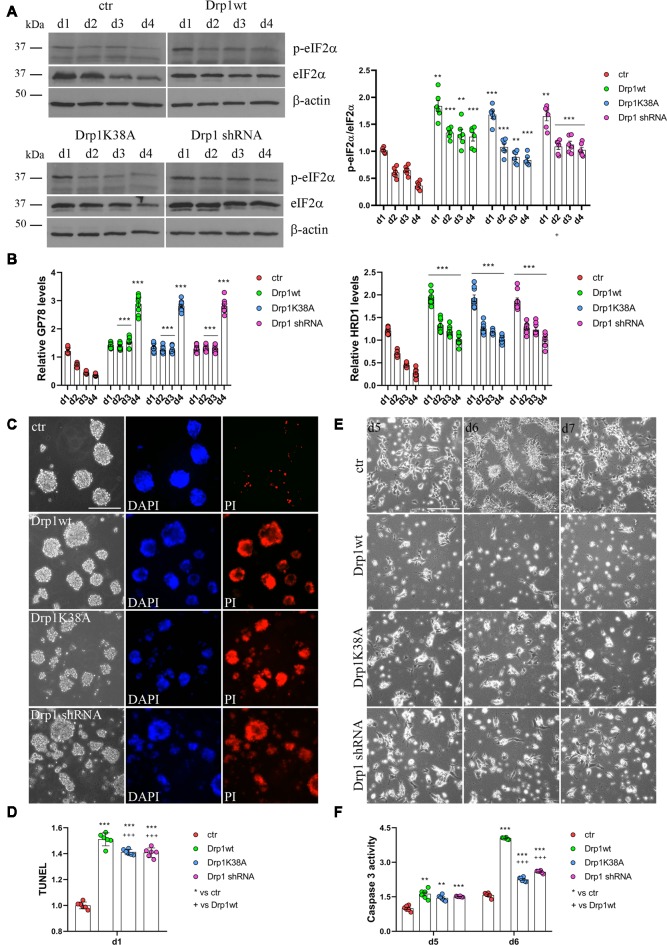
Drp1 is necessary for survival. **(A)** Drp1wt, Drp1K38A, Drp1 shRNA and control clones were induced to differentiate with RA. Total protein extracts were prepared from d1 to d4, run on 10% SDS-PAGE gels and probed with anti phospho-eIF2α (p-eIF2α) and eIF2α Abs. p-eIF2α levels were quantified, normalized on eIF2α levels and expressed as fold increase of control as individual data plus the mean ± SEM (two-way ANOVA followed by Tukey’s multiple comparison test, *n* = 6). Uncropped gels are in Supplementary Figure S12. **(B)** Drp1wt, Drp1K38A, Drp1 shRNA and control clones were induced to differentiate with RA. RNA was extracted from d1 to d4 and used to analyze GP78 and HRD1 levels by Real-Time PCR. Undifferentiated P19 cells stably transfected with the vector alone were used as endogenous control. Results are expressed as fold increase of endogenous control and reported as individual data plus the mean ± SEM (two-way ANOVA followed by Tukey’s multiple comparison test, *n* = 8). **(C)** Cell death levels in neural stem cells aggregates. Drp1wt, Drp1K38A, Drp1 shRNA and control P19 stable clones were induced to differentiate with RA and 24 h later aggregates were stained with DAPI and propidium iodide (PI) to detect living and dead cells respectively. Scale bar: 50 μm. **(D)** Morphology of differentiated cultures. Drp1wt, Drp1K38A, Drp1 shRNA and control clones were induced to differentiate with RA, on d4 were dissociated and photographed from d5 to d7. Scale bar: 50 μm. Shown are representative images. **(E)** Apoptosis levels in neural stem cells on d1 of neuronal differentiation. Drp1wt, Drp1K38A, Drp1 shRNA and control clones were induced to differentiate with RA. On d1 aggregates were dissociated and apoptosis was analyzed with TUNEL assay. TUNEL fluorescence intensity was quantified and expressed as fold increase of control. The graph shows individual data plus the mean ± SEM (one-way ANOVA followed by Tukey’s multiple comparison test, *n* = 6). **(F)** Apoptosis levels in differentiated neurons. Drp1wt, Drp1K38A, Drp1 shRNA and control clones were induced to differentiate with RA, on d4 aggregates were dissociated, plated and tested for caspase 3 activity on d5 and d6. Results shown are individual data plus the mean ± SEM (two-way ANOVA followed by Tukey’s multiple comparison test, *n* = 6). * vs ctr (***p < 0.001, **p < 0.01); ^+^ vs Drp1wt (^+++^*p* < 0.001). Lines indicate sample to whom symbols are referred.

RA induces the upregulation of several ER stress-responsive genes and ER stress play a critical role in neuronal differentiation of mouse ESCs (Liu et al., 2012; Godin et al., 2016; Murao and Nishitoh, 2017). Several stimuli activate the UPR and the ERAD pathway and both are upregulated during RA-induced differentiation (Liu et al., 2012). We analyzed UPR and ERAD pathways during neuronal differentiation (Figure 6, Supplementary Figures S12, S13) and we found that all Drp1-modified clones presented increased phosphorylation of the UPR protein eIF2α (Figure 6A and Supplementary Figure S12) compared with control and increased expression of the eIF2α downstream genes Grp78 and Grp94, the pro-apoptotic gene CHOP and of the negative regulator GADD34 (Supplementary Figure S13). Moreover also the expression levels of Gp78 and HRD1 (Figure 6B), two regulators of ERAD, were increased in Drp1-modified clones compared with control, indicating that both UPR and ERAD pathways are activated by changes in Drp1 levels.

Since Drp1 can affect cells vitality during neuronal differentiation and RA can induce apoptosis through the activation of ER stress response (Godin et al., 2016; Murao and Nishitoh, 2017), we measured cell death in neural stem cells. We used PI and DAPI to stain dead and living cells respectively (Cummings and Schnellmann, 2004), 24 h after RA induction and we found that Drp1wt, Drp1K38A and Drp1 shRNA aggregates presented higher levels of dead, PI-positive cells compared with control (Figure 6C). Apoptosis was also assessed by using a DeadEnd Fluorometric TUNEL system (Figure 6D). On d1 of neuronal differentiation, we found induction of apoptosis in all Drp1-modified clones compared to control, with however higher levels in Drp1wt when compared to Drp1K38A and Drp1 shRNA clones. No differences were observed in undifferentiated clones (Supplementary Figure S14). As a consequence of increased apoptosis and cell death, no alive cells were present in any Drp1-modified clones in late phases of neuronal differentiation and only control cells differentiated for the whole 14 days period (Figure 6E). We then analyzed apoptosis in differentiated cells quantifying the levels of caspase 3 activity on d5 and d6. While control cells presented a small increase in caspase 3 activity compared with undifferentiated cells, caspase 3 activity was significantly increased in all Drp1-modified clones, with remarkable induction in Drp1wt overexpressing clones (Figure 6F). Consistently, the levels of the activated forms of caspase 9 and caspase 7 on d5 and d6 were increased in all Drp1-modified clones compared with control (Supplementary Figure S15).

These data indicate that any Drp1 manipulation inhibits neurogenesis favoring apoptosis, regardless of mitochondrial shape.

### Physiological Levels of Drp1 Are Required for Final Neuronal Differentiation

**Figure 7:**
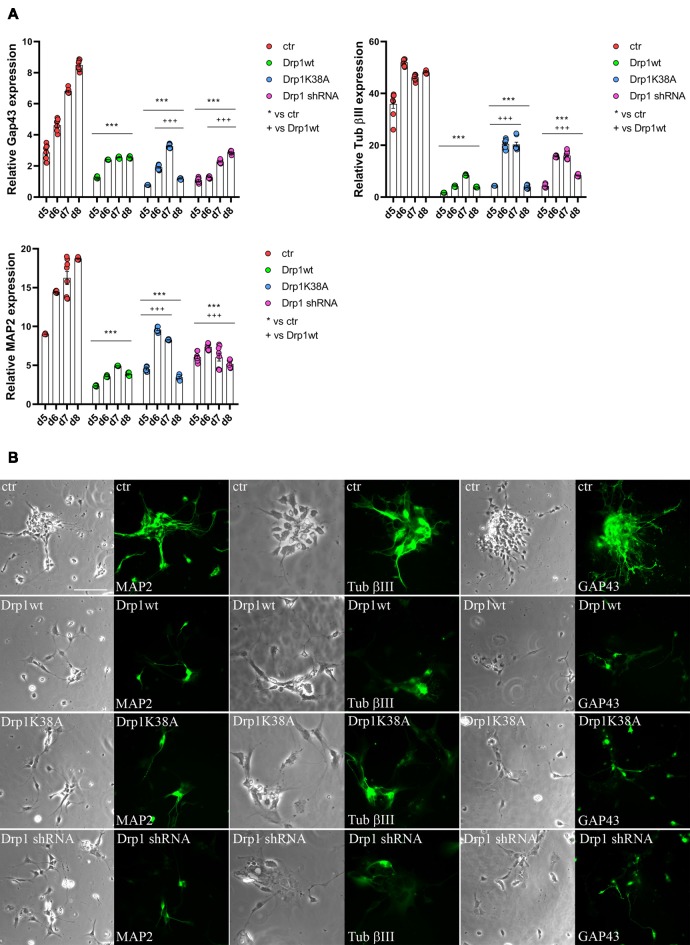
Alterations in Drp1 levels and function affect final neuronal differentiation. **(A)** Drp1wt, Drp1K38A, Drp1 shRNA and control clones were induced to differentiate with RA. RNA was extracted every day from d5 to d8 and used to analyze Tubulin β-III, MAP2 and Gap43 expression levels by Real-Time PCR. Undifferentiated P19 cells stably transfected with the vector alone were used as endogenous control. Results are expressed as fold increase of endogenous control and reported as individual data plus the mean ± SEM (two-way ANOVA followed by Tukey’s multiple comparison test, *n* = 6). * vs ctr (***p < 0.001); ^+^ vs Drp1wt (^+++^*p* < 0.001). Lines indicate samples to whom symbols are referred. **(B)** Drp1wt, Drp1K38A, Drp1 shRNA and control clones were induced to differentiate with RA and on d6 neurons were fixed and stained with anti-Tubulin β-III, MAP2 and Gap43 Abs (green). Scale bar: 50 μm.

Neuronal differentiation required both the induction of neurogenic transcription factors and the downregulation of pluripotent genes. Considering the increased levels of the pluripotent Oct3/4 and Sox2 genes and the decreased levels of the RA-induced genes Mash1, Wnt1 and N-cadherin we detected in all Drp1-modified clones, we assessed the formation of neurons. The expression levels of Tubulin β-III, Gap43 and MAP2, markers respectively of neurofilaments, growth cones and neurites, were significantly reduced in all Drp1-modified clones compared to control (Figure 7A), however with a more severe effect of Drp1wt overexpression. In agreement, while in control cells the 90% of differentiated cells were positive for neuronal markers and showed normal development of neuronal processes and extensive connections, as described (Bain et al., 1994), only a few cells in Drp1-modified clones were positive for Tubulin β-III, Gap43 and MAP2 (Figure 7B).

## Discussion

The importance of mitochondrial dynamics in neurons and brain physiology has become increasingly clear. Neurons require high levels of energy for survival and for their specialized functions. These highly polarized cells are particularly vulnerable to mitochondrial fission and fusion defects that lead to deficient cell bioenergetics and incorrect distribution of mitochondria along the axons and at the synapses. At these sites, high levels of ATP are required for transmission and for synapses organization (Li et al., 2004, 2008; Chen and Chan, 2006). While Drp1-dependent mitochondrial fission seems to be dispensable for the function of non-polarized cells, this function is required for extremely polarized cells such as neurons. Many studies dissected the physiological role of mitochondrial fission in neurons evaluating the effects of Drp1 deletion. Drp1 is required for the development of the mammalian nervous system (Waterham et al., 2007; Ishihara et al., 2009; Wakabayashi et al., 2009) and Drp1 deficiency results in depletion of mitochondria from developing neurites, reduced neurite outgrowth, impaired synapse formation (Ishihara et al., 2009) finally leading to neurodegeneration (Kageyama et al., 2012, 2014). Drp1 ablation alters neuronal functions also in adult neurons, affecting synaptic transmission and memory, however, compensatory circuits are sufficient to maintain overall neuronal morphology and viability (Oettinghaus et al., 2016). Moreover, Drp1 hyper-phosphorylation at Ser-637 induces abnormal mitochondrial elongation and is associated with hereditary spastic paraplegia (Lavie et al., 2017). The reduction in Drp1-mediated fission compromises mitochondrial health in spastic ataxia of Charlevoix Saguenay (Bradshaw et al., 2016). All these findings describe the effects of extensively fused mitochondrial structure. Conversely, a wide range of neurodegenerative disorders, such as Alzheimer’s (Cho et al., 2009; Manczak et al., 2011; Manczak and Reddy, 2012), Huntington’s (Costa et al., 2010; Song et al., 2011; Shirendeb et al., 2012) and Parkinson’s diseases (Wang et al., 2011) are associated with increased Drp1 levels and mitochondrial fragmentation (Hu et al., 2017). The biological significance of the increased Drp1 levels in neuronal differentiation was not investigated. We, therefore, analyzed the effects of increased levels of Drp1 on P19 cell neuronal differentiation, comparing the phenotype of Drp1 overexpression to that of the well-known Drp1 deletion. We demonstrate here that P19 cell neuronal differentiation is associated with key changes in mitochondrial fission and fusion genes. In particular, Drp1 levels gradually increased during RA treatment to reach 2.5–3-fold increase in differentiated neurons, confirming the reported augment of Drp1 observed in the late stage of ESCs differentiation (Wang et al., 2014). This Drp1 expression pattern is paralleled by enhanced Mfn1/2 expression and both contribute to mitochondrial remodeling, with prevalently small and round mitochondria in undifferentiated cells that become elongated in differentiated neurons. These data are also in agreement with the metabolic shift observed in undifferentiated P19 cells that are essentially glycolytic, while differentiated neurons are more oxidative (Vega-Naredo et al., 2014). We genetically manipulated Drp1 generating P19 stable clones overexpressing Drp1wt, Drp1K38A or silencing the gene, and, differently from previous studies, we monitored neuronal differentiation. Neuronal differentiation is characterized by the induction of RA-dependent neurogenic transcription factors, and by the downregulation of pluripotency genes, such as Oct3/4 and Sox2. All Drp1-modified clones shared similarities in the response to RA showing lower levels of the RA-induced genes Mash1, Wnt1 and N-cadherin and failed to downregulate Oct3/4 and Sox2 expression, suggesting that mitochondrial changes can induce a transcriptional reprogram that negatively impacts on neuronal differentiation. Sox2 is expressed in multipotent neuronal stem cells and is downregulated during differentiation. Here, we found that Sox2 expression was not downregulated in Drp1-modified clones during induction of neuronal differentiation and was still high on d5–d7, inhibiting final neuronal differentiation. Consistently, we found that the concomitant reduction in the expression of neurogenic transcription factors and the increased levels of pluripotency genes in Drp1wt, Drp1K38A and Drp1-silenced clones, correlated with a defective differentiation with decreased expression of Tubulin β-III, MAP2 and Gap43 neuronal markers in mature neurons. Indeed, Sox2 is mutually exclusive with Tubulin β-III marker and its constitutive expression inhibits neuronal differentiation in CNS and results in the maintenance of progenitor characteristics (Graham et al., 2003).

Our data indicate that these defects occur in a mitochondrial-dependent manner. During neuronal differentiation, control clones undergo mitochondrial remodeling characterized by increased elongation, branch length and interconnectivity. Autophagy induction during the first phases of differentiation is likely to concur to this specific mitochondrial network organization and both events allow correct neurogenesis. This is common to other differentiation processes, such as myogenesis, in which Drp1-mediated fission appears to be crucial for mitophagy. Following the mitochondrial clearance stage, mitochondria are substituted and form a more organized network and this rebuilding phase does not occur until mitochondrial clearance takes place (Sin et al., 2016).

High Drp1 levels specifically increase mitochondrial fission with less organized and more fragmented mitochondria that are able to undergo remodeling during neuronal differentiation, increasing interconnectivity and branch length, but fails to elongate, thus definitely impairing neurogenesis. Consistently, autophagy and apoptosis are extremely enhanced, preventing differentiation. Both the Drp1K38A overexpression and Drp1 silencing enhance mitochondria elongation, interconnectivity and branch length in undifferentiated conditions, likely due to inhibition of fission and unopposed fusion, as supported by unchanged MFNs expression and identical Opa1 activity, similarly to other models (Kageyama et al., 2012, 2014). Also, Drp1K38A overexpression and Drp1 silencing prevent the required mitochondrial remodeling, leading to increased apoptosis and neuronal death.

Inhibition of mitochondrial respiration and increased ROS production affect gene expression resulting in the impairment of differentiation capability and the enhancement of stem cell pluripotency (Varum et al., 2009). Accordingly, inefficient oxidative metabolism increases ROS production and prevents P19 cells neuronal differentiation (Pashkovskaia et al., 2018). In line with this, our clones, regardless of their mitochondria shape, displayed reduced mitochondrial membrane potential and bioenergetic defects, leading to inefficient neuronal differentiation.

We demonstrate that neuronal differentiation is dramatically susceptible to abnormal mitochondrial fission and high Drp1 expression and not only to the complete absence of the protein, suggesting that physiological fission and mitochondrial remodeling, associated with early autophagy induction, are essential for neuronal differentiation. Any unbalance of mitochondrial remodeling affects autophagy, apoptosis or both, thereby impairing neuronal differentiation. Considering that neither the FGF8 activated pathways, i.e., ERK1/2 and Akt, nor BMP4 pathway, through Smad1 inhibition, are affected in Drp1-modified clones, we can conclude that increased apoptosis could derive from altered RA response, likely through hyperactivation of UPR and ERAD pathways. Indeed, while a progressive increase in UPR is required for normal neuronal differentiation, prolonged UPR activation can induce apoptosis and cell death (Godin et al., 2016; Murao and Nishitoh, 2017).

The link between mitochondrial fission and apoptosis is not surprising (Itoh et al., 2013) and also the knockdown and the dominant-negative interference of endogenous Drp1 have been demonstrated to cause cell death in cortical neurons (Uo et al., 2009; Bradshaw et al., 2016), indicating a high vulnerability of neurons to alterations in Drp1 levels and activity. Moreover, Drp1 has been reported to induce mitochondrial damage and apoptosis independently of its GTPase activity (Bras et al., 2007).

In summary, we found that neuronal differentiation is susceptible to changes in Drp1 levels and function, and it is severely impaired by both Drp1 overexpression and protein depletion or inactivation. This suggests the existence of a relationship between mitochondrial dynamics and neurodegeneration involving both fission induction and suppression. This appears to be of broad significance as strategies normalizing mitochondrial dynamics appear to be effective for a wide range of neurodegenerative diseases (Guo et al., 2013; Franco et al., 2016; Joshi et al., 2018).

Finally, our study provides novel information on the role of Drp1 in neuronal differentiation induction adding hints to the knowledge regarding the effects of Drp1 depletion in post-mitotic neurons. This study thus reveals the importance of mitochondrial changes at the onset of neuronal differentiation, sharing similarity with other stem cells and differentiation pathways, and highlights that, rather than multiple parallel events, mitochondrial changes, autophagy and apoptosis proceed in a stepwise fashion during neuronal differentiation altering the nuclear transcriptional program.

## Author Contributions

CV analyzed, differentiated and undifferentiated Drp1 clones, performed neuronal differentiation, autophagy and cell death analysis, immunofluorescence, western-blot and statistical analysis, designed the experiments, interpreted the data and wrote the manuscript. MC prepared Drp1 stable clones, performed neuronal differentiation and analyzed gene expression levels. MG performed mitochondrial fractionation, mtDNA analysis and mitochondrial membrane potential quantification. GO performed immunofluorescence, confocal analysis and mitochondrial morphometric analysis. MB and EC interpreted the data, supervised the research and critically revised the manuscript. CDP designed the experiments, interpreted the data and wrote the manuscript.

## Conflict of Interest Statement

The authors declare that the research was conducted in the absence of any commercial or financial relationships that could be construed as a potential conflict of interest.

## Footnotes

^1^www.graphpad.com

## Abbreviations

Drp1: dynamin-related protein 1
eIF2α: eukaryotic translation initiation factor 2 alpha
ERAD: ER-associated degradation
ESCs: embryonic stem cells
Fis1: fission 1
Mff: mitochondrial fission factor
Mfn1: mitofusins 1
Mfn2: mitofusins 2
MiD: mitochondrial dynamics proteins
mtCO1: mitochondrial Cytochrome C Oxidase I
mtDNA: mitochondrial DNA
Opa1: optic atrophy 1 protein
RA: retinoic acid
TMRM: tetramethylrhodamine methyl ester
UPR: unfolded protein response
VDAC: voltage-dependent anion channels.
