# Supplementary material for: The Fine Tuning of Drp1-Dependent Mitochondrial Remodeling and Autophagy Controls Neuronal Differentiation
Source: Front Cell Neurosci. 2019 Apr 4;13:120. doi: 10.3389/fncel.2019.00120 (PMC6458285; doi:10.3389/fncel.2019.00120)
Supplement: Supplementary file 1 [file Data_Sheet_1.pdf]

Supplementary Material

The fine tuning of Drp1-dependent mitochondrial remodeling and autophagy controls neuronal differentiation

Chiara Vantaggiato[1,*], Marianna Castelli[1], Matteo Giovarelli[2], Genny Orso[3], Maria Teresa Bassi[1], Emilio Clementi[1,2], Clara De Palma[4]

Correspondence
Corresponding Author: Chiara Vantaggiato PhD, e-mail chiara.vantaggiato@lanostrafamiglia.it.

Supplementary Figures

Supplementary Figure 1

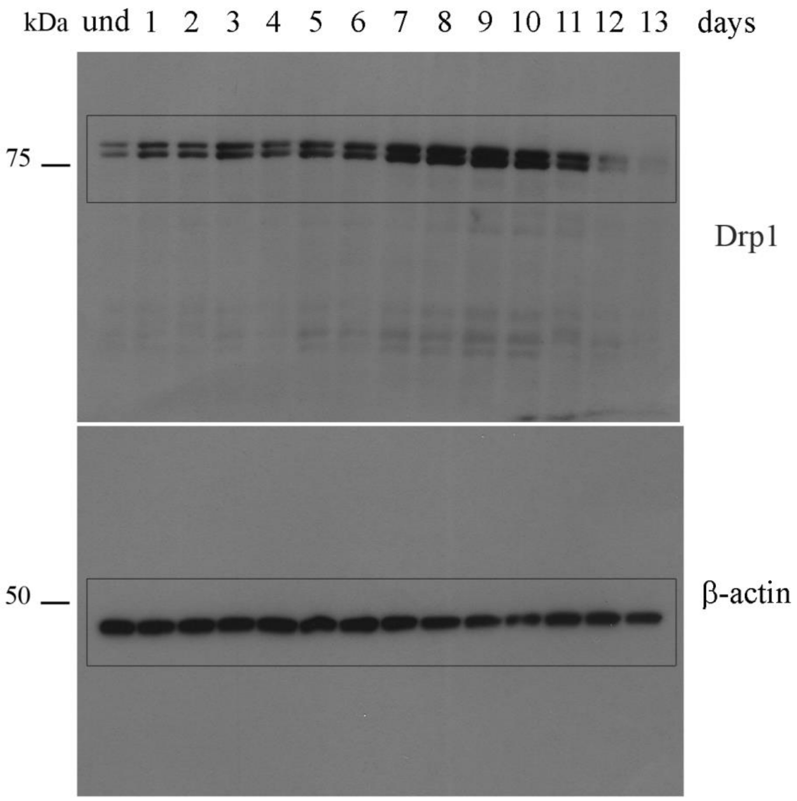

Supplementary Figure 1. Uncropped gel of Fig. 1C.

Supplementary Figure 2

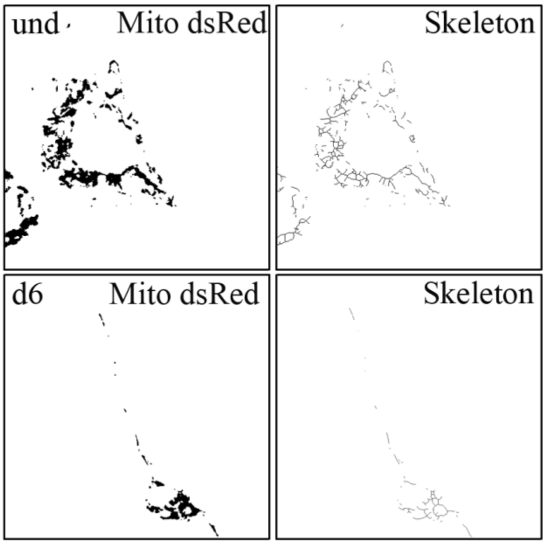

Supplementary Figure 2. Binary and skeleton images of Fig. 1E. Undifferentiated control cells and neurons on d5 of neuronal differentiation were transfected with the pDsRed2-Mito vector for the staining of mitochondria and fixed after 24h. Nuclei were stained with DAPI. Images were acquired by confocal microscopy and morphometric analysis was performed with ImageJ. Red channels were converted into a black binary image and skeletonized (skeleton).

Supplementary Figure 3

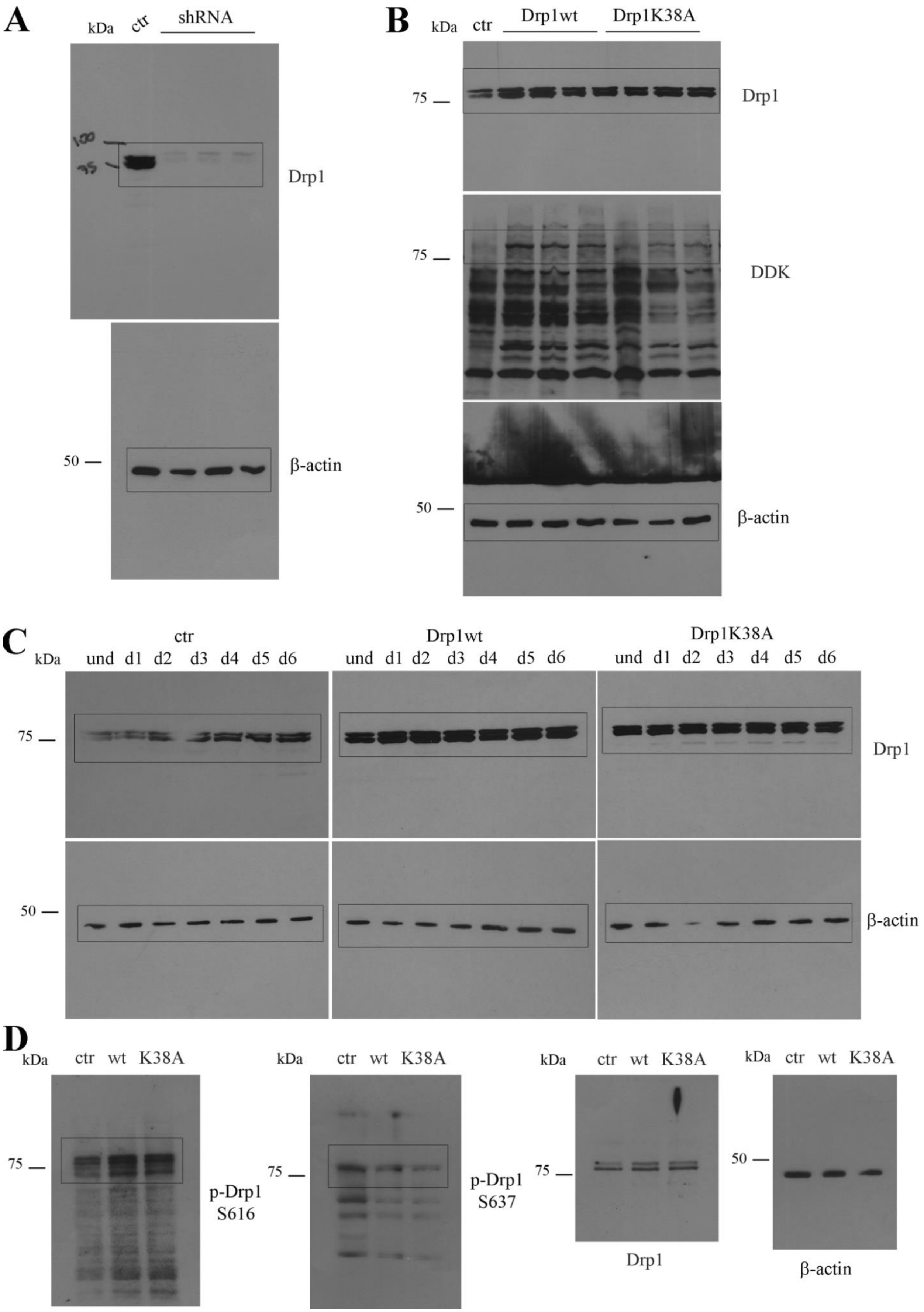

Supplementary Figure 3. Uncropped gels of Fig.2B (**A**), Fig. 2C (**B**), Fig. 2D (**C**) and Fig. 2F (**D**).

Supplementary Figure 4

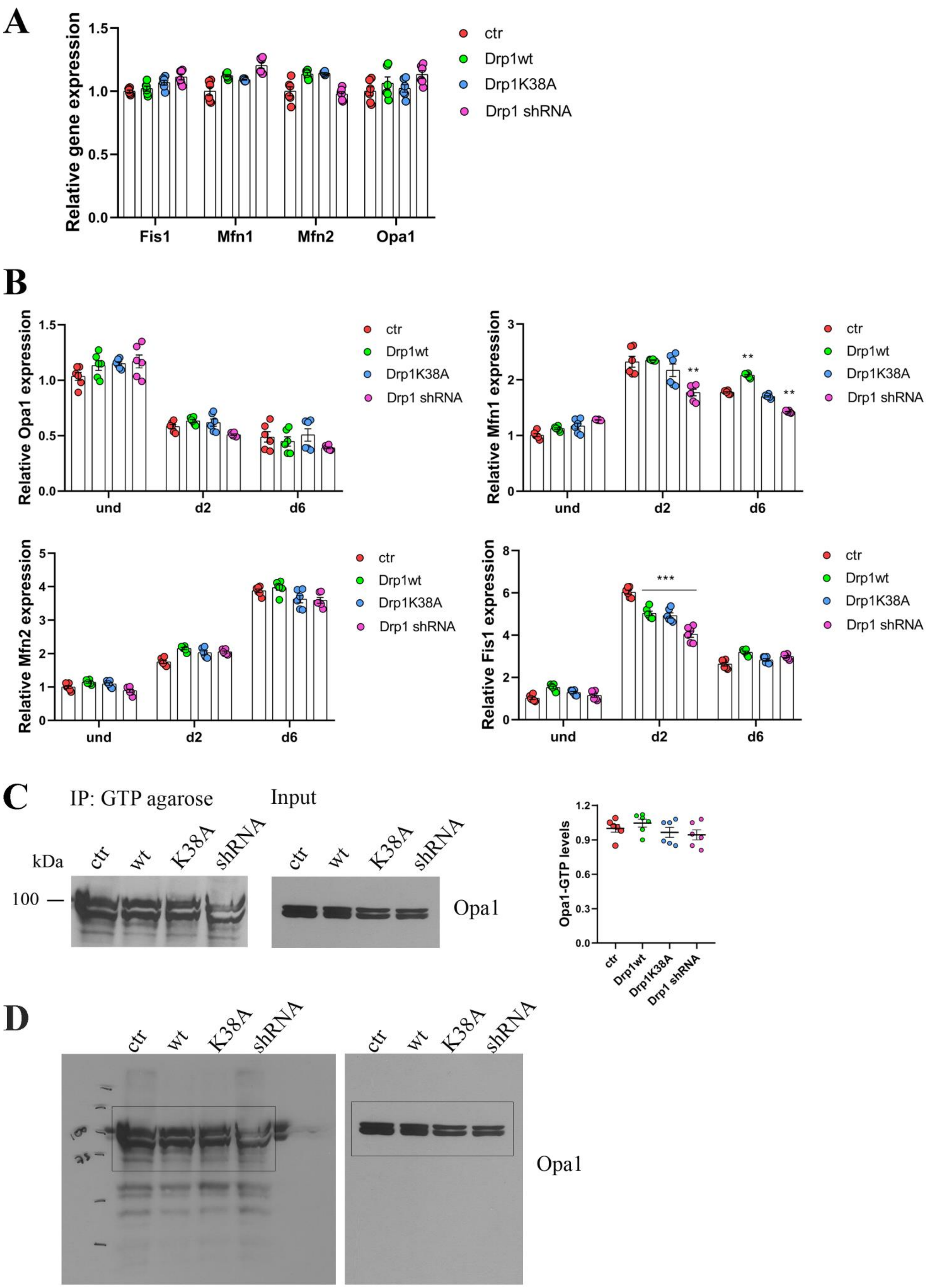

Supplementary Figure 4. (**A**) Fission and fusion gene expression in undifferentiated clones. RNA was extracted from undifferentiated Drp1wt, Drp1K38A, Drp1 shRNA and control clones and used to analyze the expression of Fis1, Mfn1, Mfn2 and Opa1 by Real Time PCR. Results are expressed as fold increase of undifferentiated control cells, used as endogenous control, as individual data plus the mean ± SEM (two-way ANOVA followed by Tukey's multiple comparison test, n=6). (**B**) Drp1 does not affect fission and fusion gene expression during differentiation. RNA from undifferentiated Drp1wt, Drp1K38A, Drp1 shRNA and control clones and from differentiated cells on d2 and d6 was used to analyse the expression levels of Opa1, Mfn1, Mfn2 and Fis1 by Real Time PCR. Results are expressed as fold increase of undifferentiated control cells, used as endogenous control, as individual data plus the mean ± SEM (two-way ANOVA followed by Tukey's multiple comparison test, n=6). * vs ctr (*** $p<0.001$, ** $p<0.01$). (**C**) Opa1 activity in undifferentiated clones. Total extracts from Drp1-modified and control clones were incubated with GTP-agarose beads, loaded on a 10% SDS-polyacrilamide gel and incubated with anti Opa1 Ab. Opa1-GTP levels were quantified, normalized on input levels and showed in the graph as indivial data plus means ± SEM (one-way ANOVA followed by Dunnett's multiple comparison test, n=6). (**D**) Uncropped gels of panel C.

Supplementary Figure 5

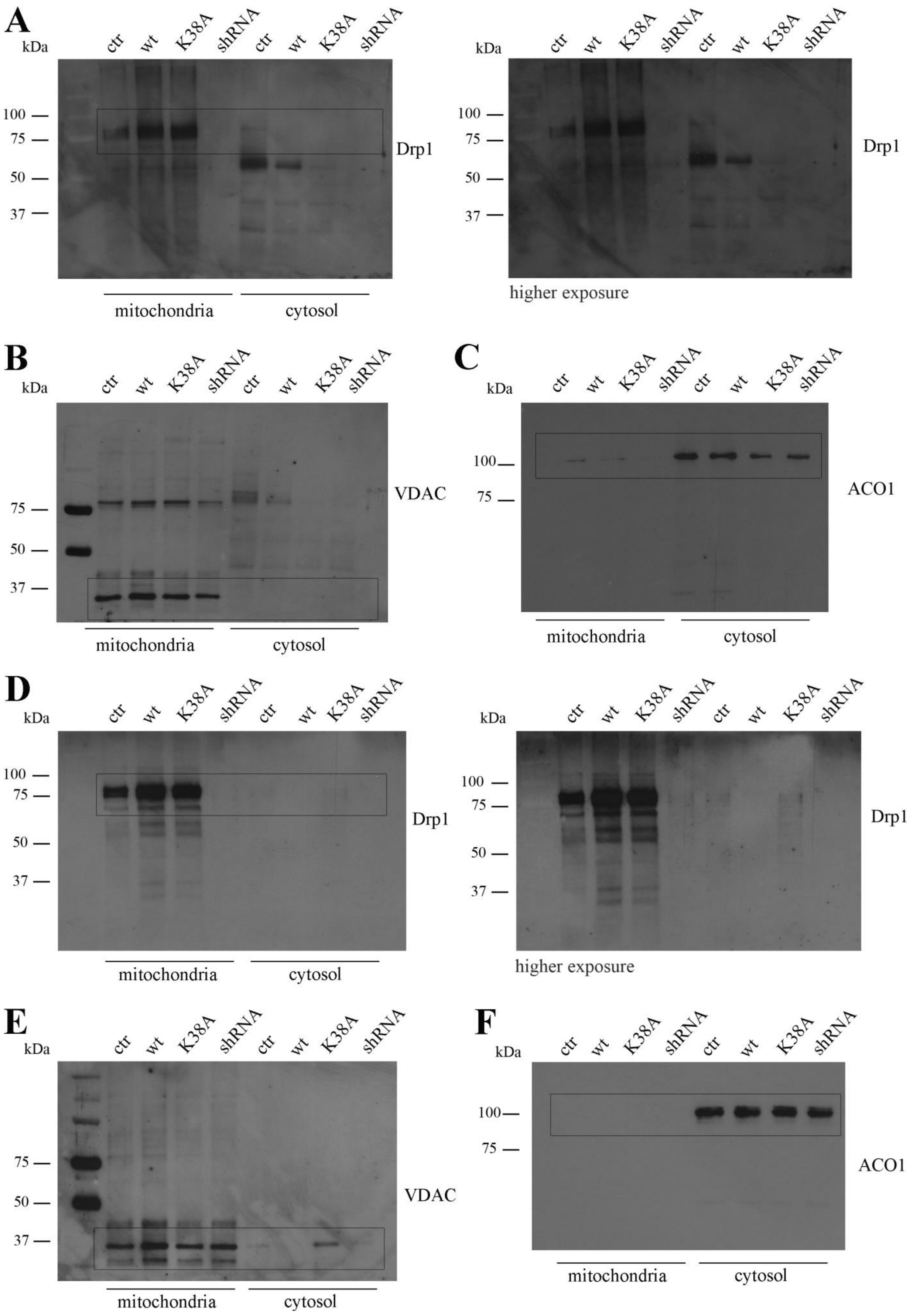

Supplementary Figure 5. (**A**)(**B**)(**C**) Original uncropped gels of Fig. 2E and (**D**)(**E**)(**F**) of Fig. 2G.

Supplementary Figure 6.

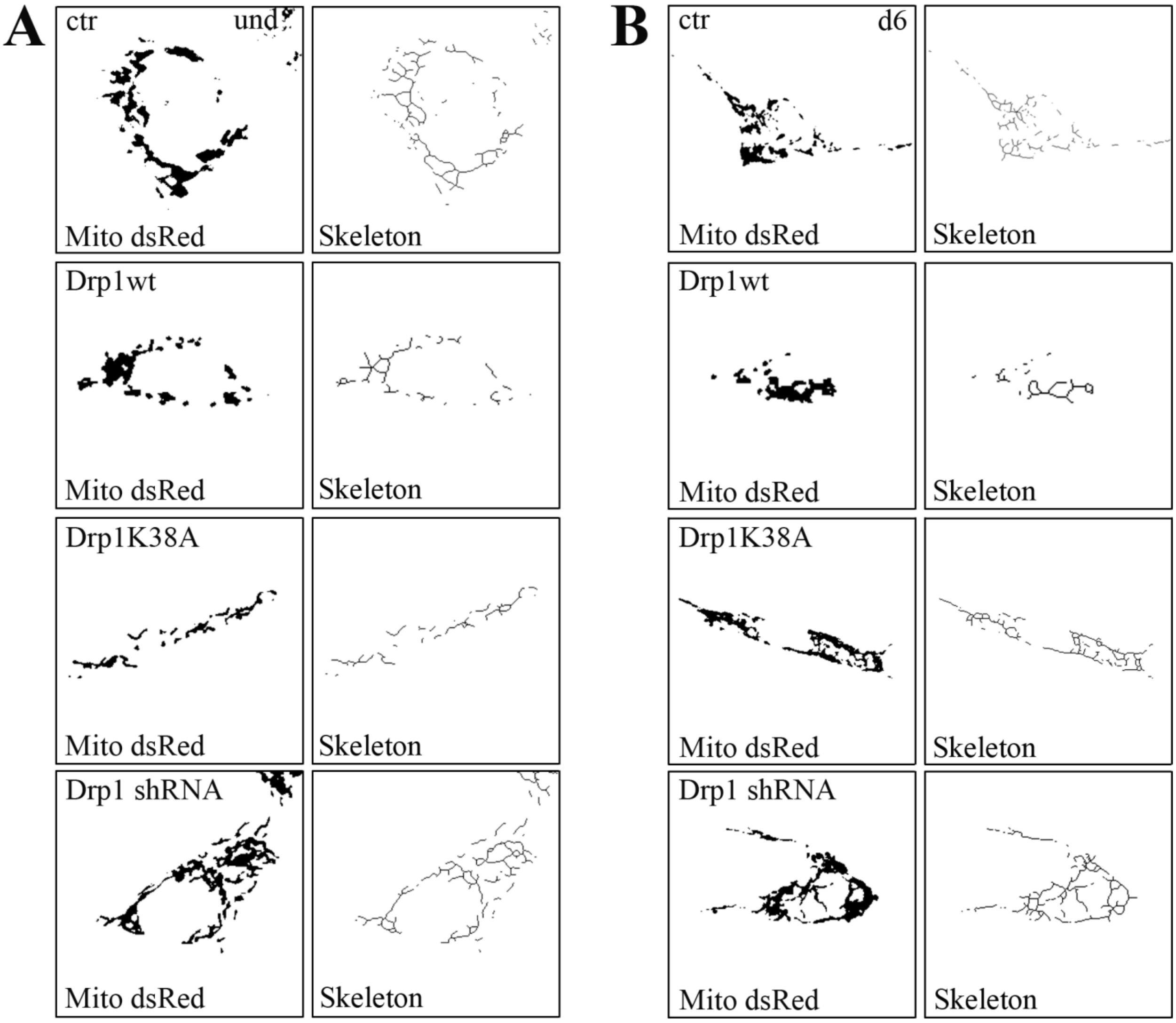

Supplementary Figure 6. Binary and skeleton images of Fig. 3. (**A**) Binary and skeleton images of undifferentiated control and Drp1-modified clones of Fig. 3A. (**B**) Binary and skeleton images of control and Drp1-modified clones of Fig. 3B on d6 of neuronal differentiation.

Supplementary Figure 7

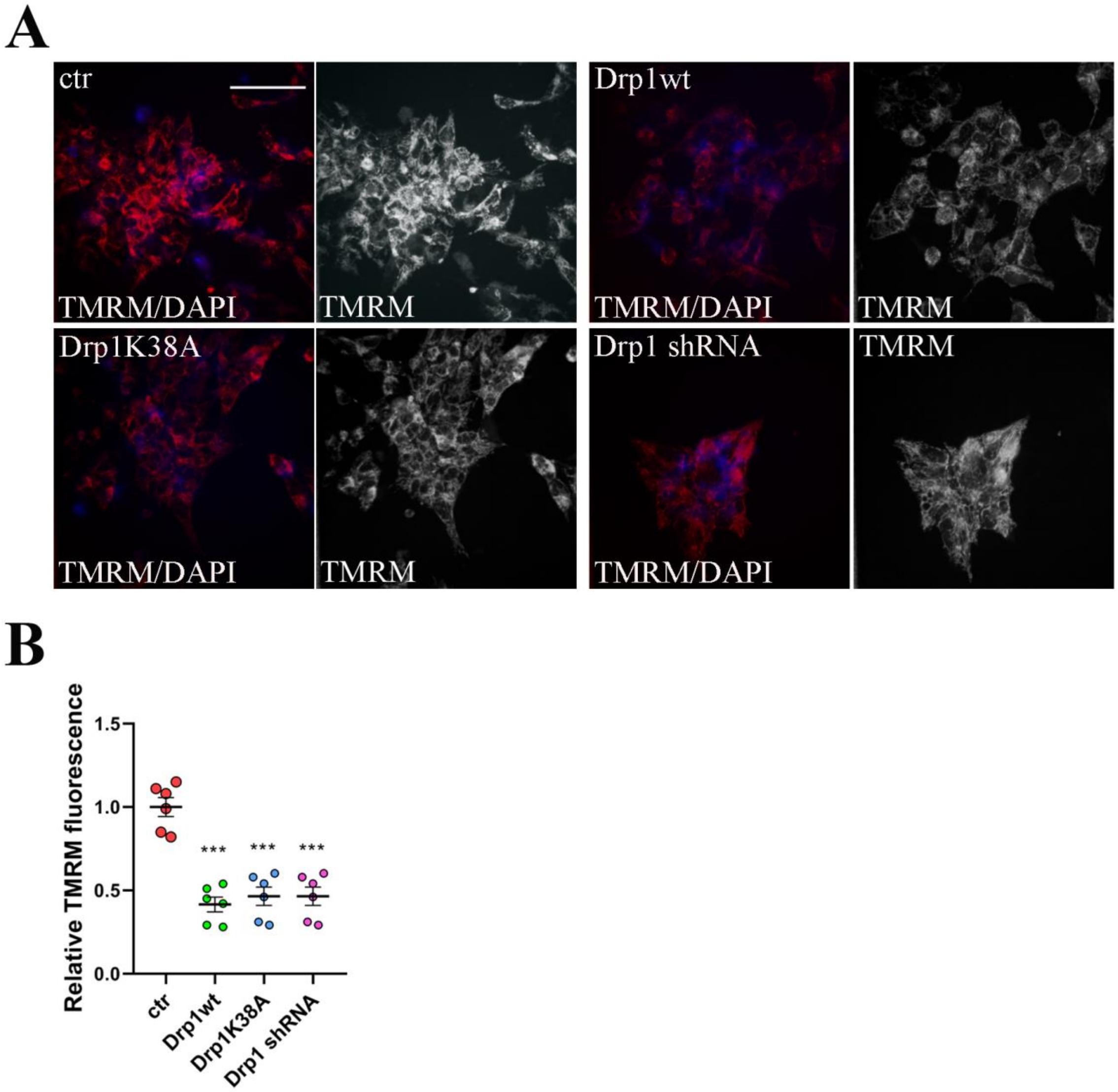

Supplementary Figure 7. Mitochondrial membrane potential. (**A**) Undifferentiated Drp1wt, Drp1K38A, Drp1 shRNA and control cells were incubated with 100 nM TMRM and 1 µg/ml DAPI for 30 min at 37°C. Image were acquired with confocal microscope at the same laser attenuation. Scale bar: 100 µm. (**B**) TMRM fluorescence intensity was quantified and expressed as fold increase of control as individual data plus the mean ± SEM (one-way ANOVA followed by Dunnett's multiple comparison test, n=6).* vs ctr (*** $p<0.001$).

Supplementary Figure 8

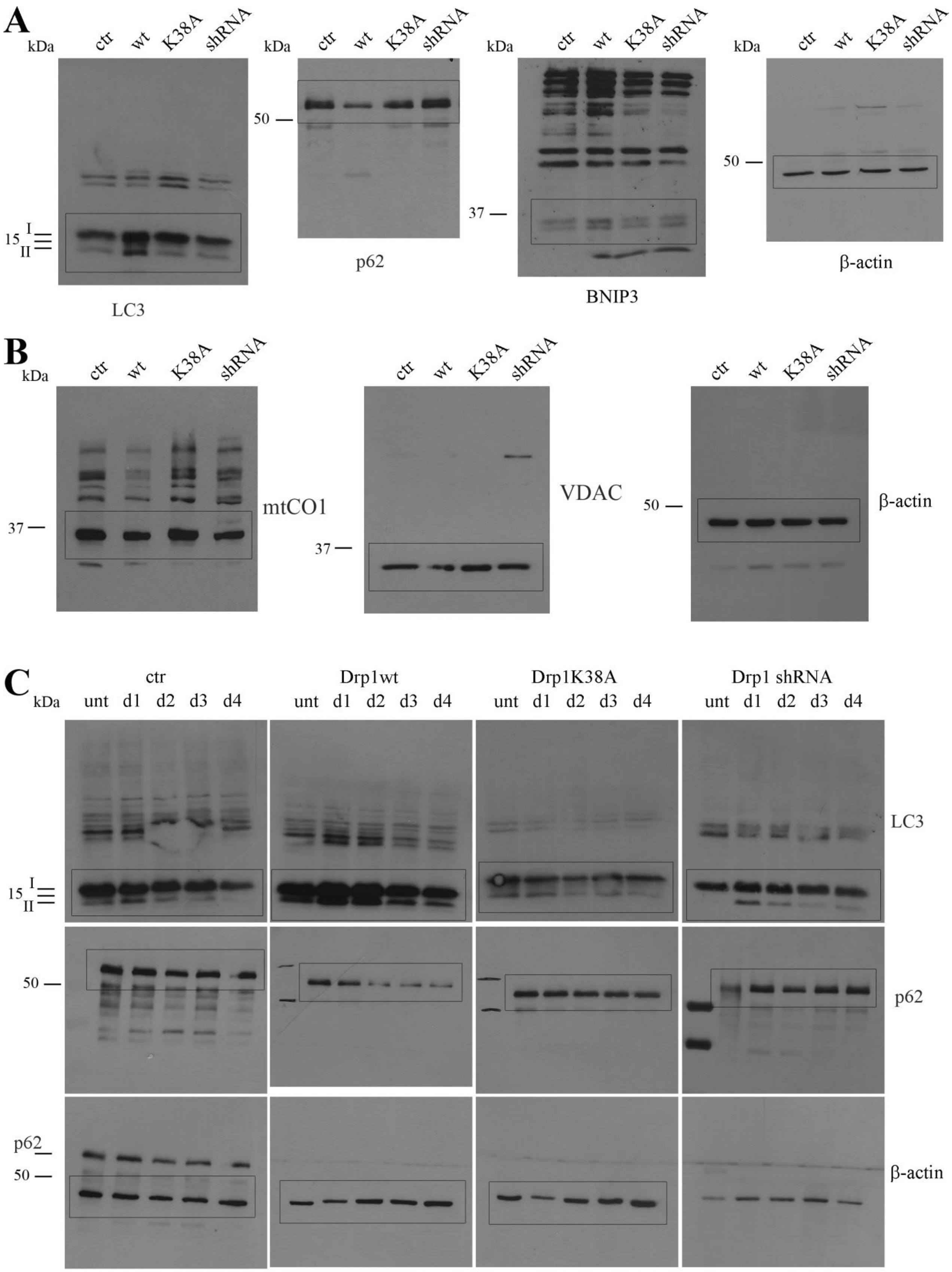

Supplementary Figure 8. Uncropped gels of Fig. 4A (**A**), Fig.4E (**B**) and Fig. 4F (**C**).

Supplementary Figure 9

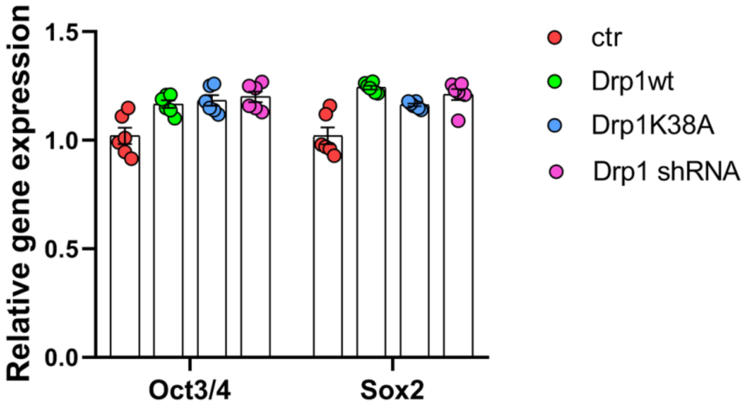

Supplementary Figure 9. Oct3/4 and Sox2 expression levels in undifferentiated clones. RNA was extracted from undifferentiated Drp1wt, Drp1K38A, Drp1 shRNA and control clones and used to analyze the expression of Oct3/4 and Sox2 by Real Time PCR. Results are expressed as fold increase of undifferentiated control cells, used as endogenous control, as individual data plus means ± SEM (one-way ANOVA followed by Sidak's multiple comparison test, n=6).

Supplementary Figure 10

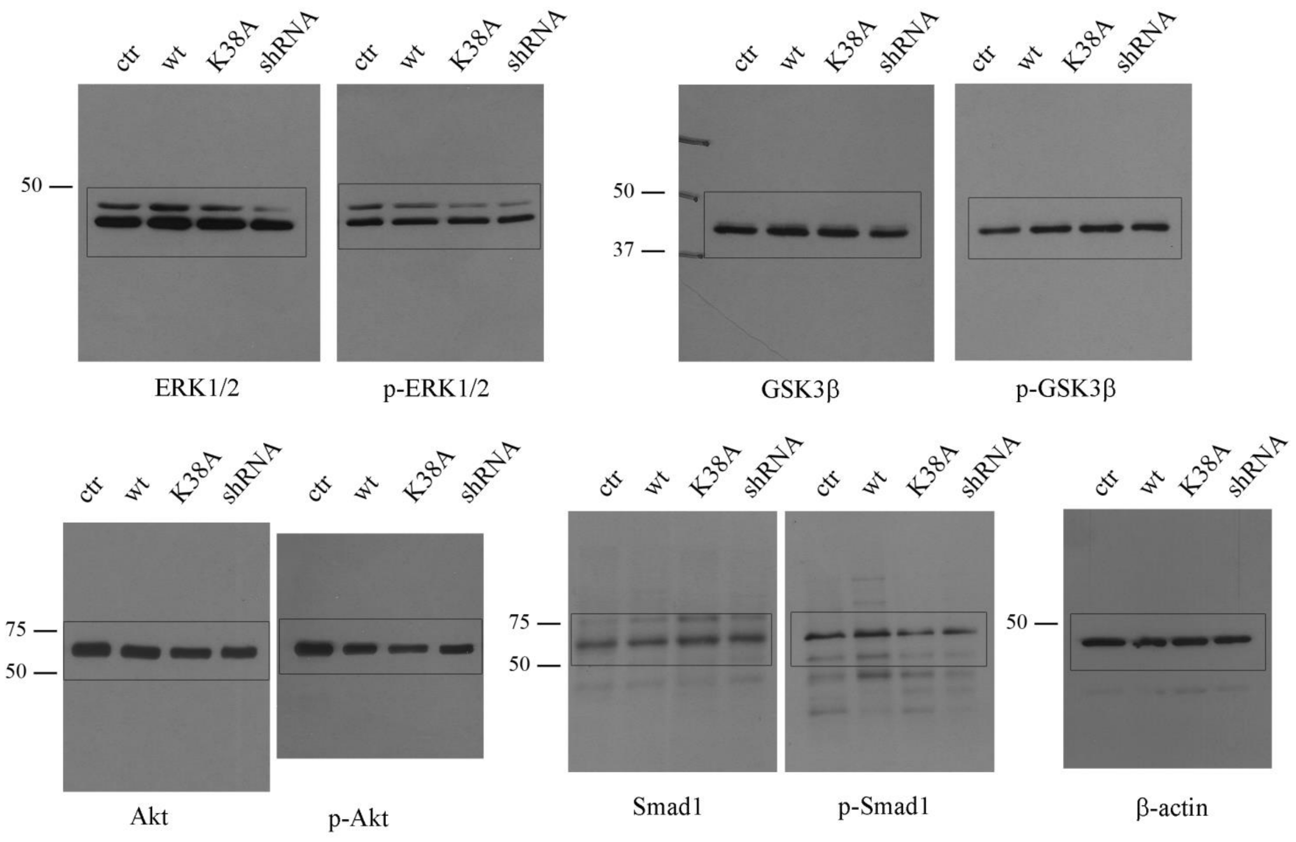

Supplementary Fig. 10. Uncropped gels of Fig. 5D.

Supplementary Fig. 11

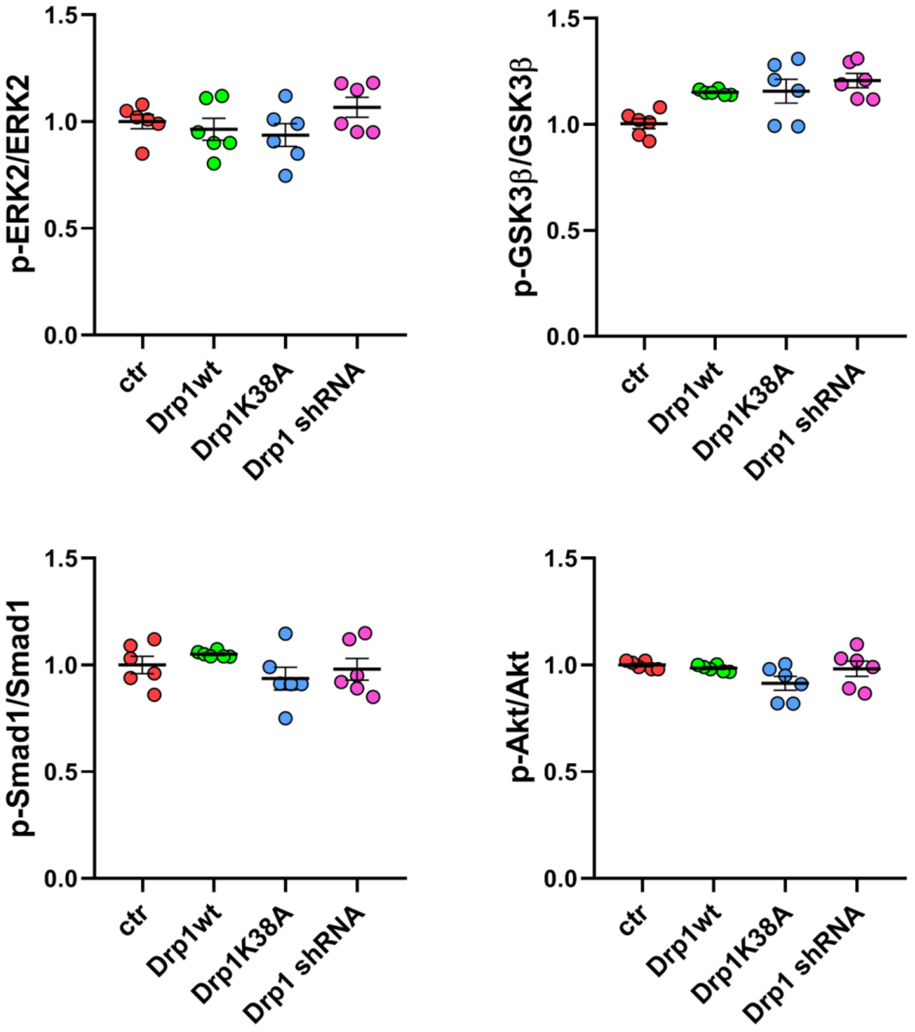

Supplementary Figure 11. Quantification of gels of Figure 5D. Phosphorylation levels of Akt, GSK3β, ERK2 and Smad1 were quantified from the gels of Fig. 5D and normalized on total Akt, GSK3β, ERK2 and Smad1. The graphs show individual data plus the mean ± SEM (one-way ANOVA followed by Dunnett's multiple comparison test, n=6).

Supplementary Figure 12

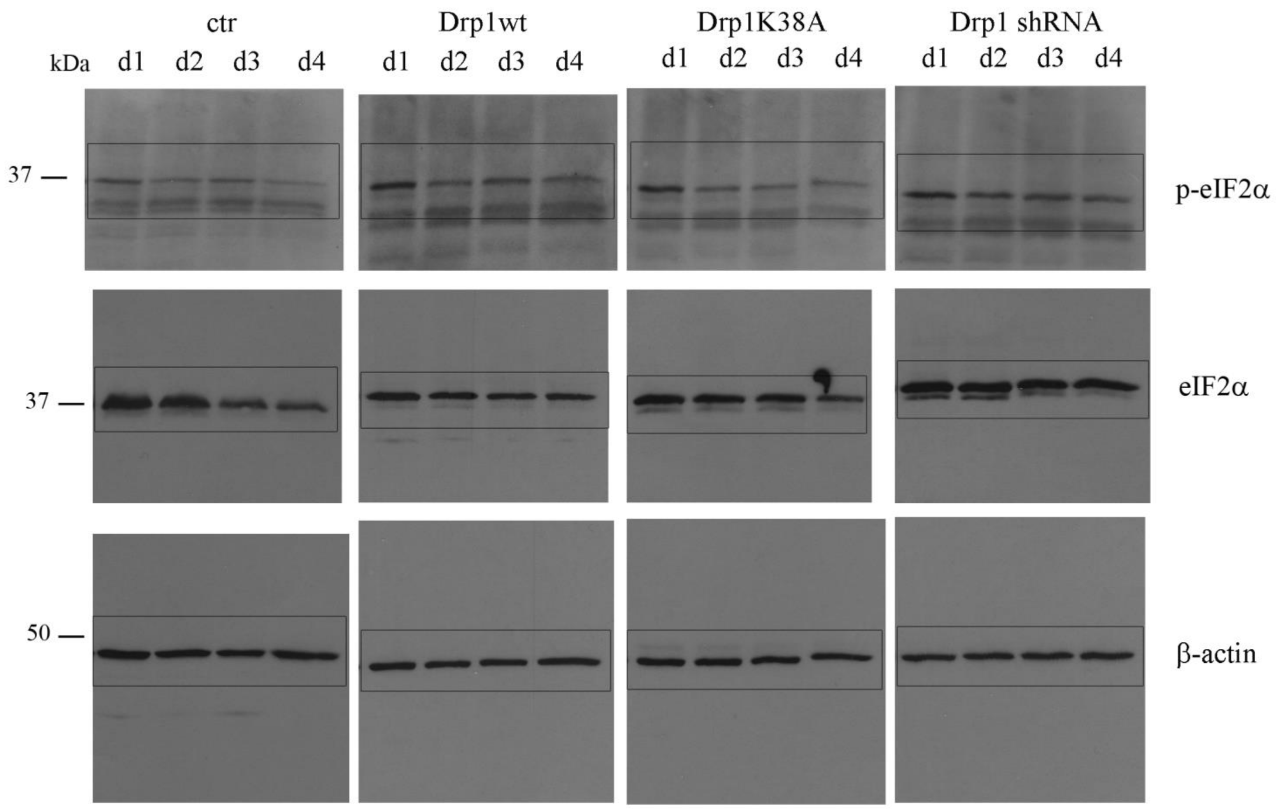

Supplementary Figure 12. Uncropped gels of Fig. 6A.

Supplementary Fig. 13

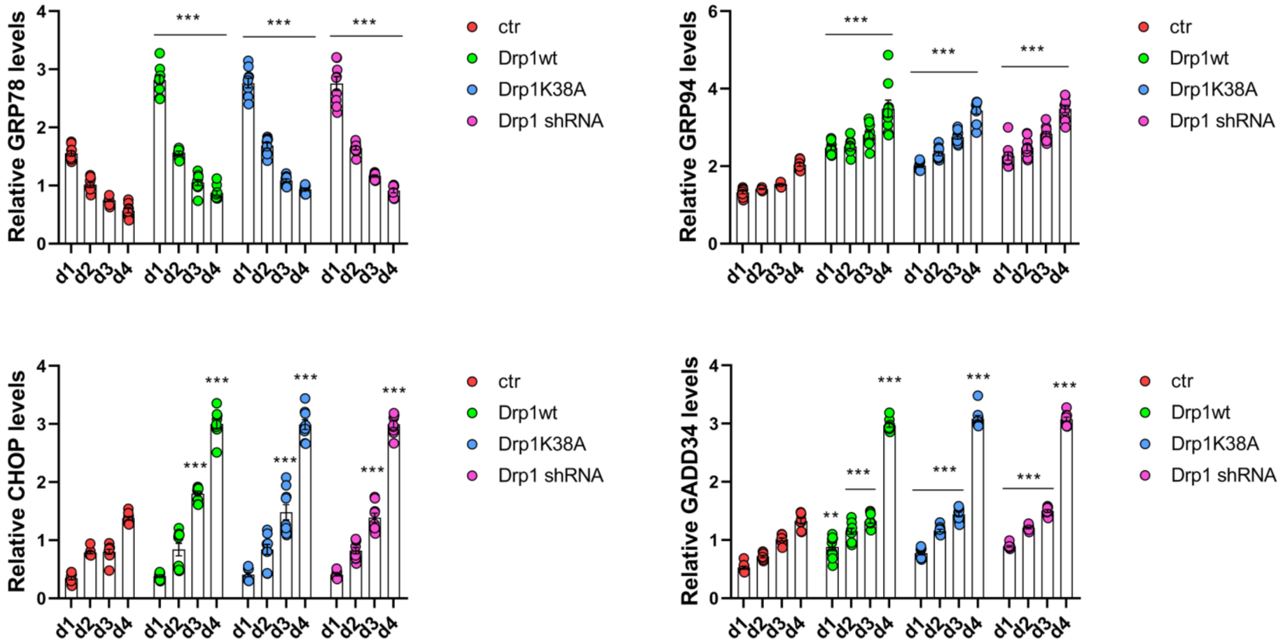

Supplementary Figure 13. GRP78, GRP94, CHOP and GADD34 expression levels. Drp1wt, Drp1K38A, Drp1 shRNA and control clones were induced to differentiate with RA. RNA was extracted from d1 to d4 and used to analyze GRP78, GRP94, CHOP and GADD34 expression levels by Real Time PCR. Undifferentiated control cells were used as endogenous control. The graphs show individual data plus the mean ± SEM (two-way ANOVA followed by Tukey's multiple comparison test, n=6) * vs ctr (*** $p<0.001$). Lines indicates samples to whom the asterisks are referred.

Supplementary Figure 14

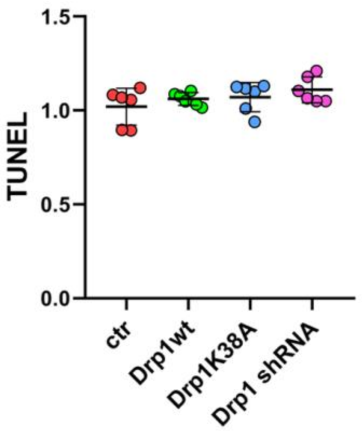

Supplementary Figure 14. Apoptosis was analyzed in undifferentiated clones with TUNEL assay. TUNEL fluorescence intensity was quantified and expressed as fold increase of control. The graph shows individual data plus the mean ± SEM (one-way ANOVA followed by Dunnett's multiple comparison test, n=6).

Supplementary Figure 15

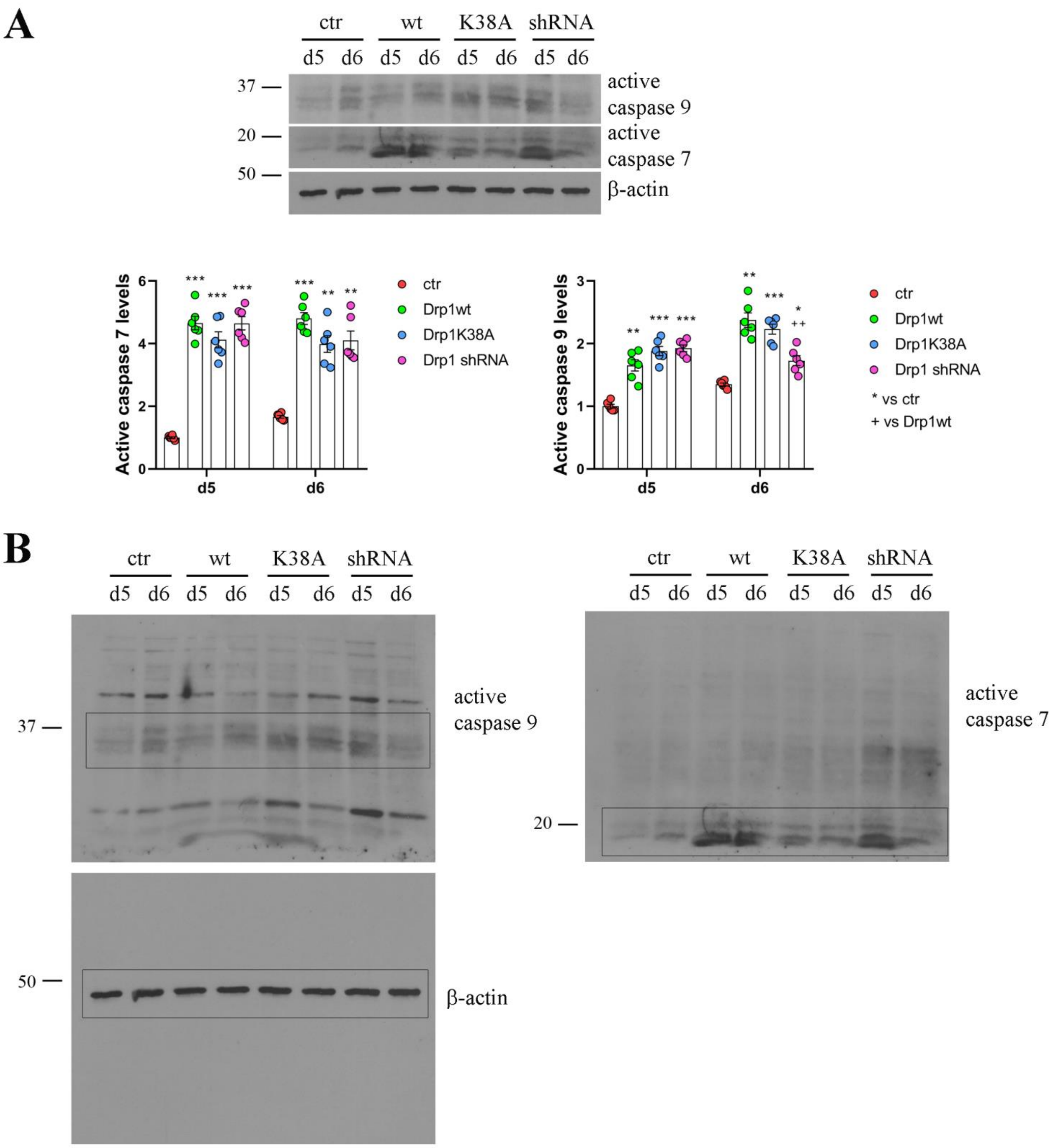

Supplementary Figure 15. Active caspase 9 and caspase 7 levels. (**A**) Total extracts were prepared from Drp1wt, Drp1K38A, Drp1 shRNA and control clones on d5 and d6 of neuronal differentiation, run on a 10% SDS-PAGE gel and probed with anti-active caspase 9 and active caspase 7 Abs. Relative bands were quantified, normalized on β-actin levels and expressed as fold increase of

control. The graphs show individual data plus the mean ± SEM (two-way ANOVA followed by Tukey's multiple comparison test, n=6). * vs ctr (* $p < 0.05$, ** $p < 0.01$, *** $p < 0.001$); + vs Drp1wt (++ $p < 0.01$). (**B**) Uncropped gels of panel A.
